# Design and quality control of large-scale two-sample Mendelian randomization studies

**DOI:** 10.1093/ije/dyad018

**Published:** 2023-04-12

**Authors:** Philip C Haycock, Maria Carolina Borges, Kimberley Burrows, Rozenn N Lemaitre, Sean Harrison, Stephen Burgess, Xuling Chang, Jason Westra, Nikhil K Khankari, Kostas K Tsilidis, Tom Gaunt, Gibran Hemani, Jie Zheng, Therese Truong, Tracy A O’Mara, Amanda B Spurdle, Matthew H Law, Susan L Slager, Brenda M Birmann, Fatemeh Saberi Hosnijeh, Daniela Mariosa, Christopher I Amos, Rayjean J Hung, Wei Zheng, Marc J Gunter, George Davey Smith, Caroline Relton, Richard M Martin, Fatty Acids in Cancer Mendelian Randomization Collaboration, Nathan Tintle, Ulrike Peters, Terri Rice, Iona Cheng, Mark Jenkins, Steve Gallinger, Alex J. Cornish, Amit Sud, Jayaram Vijayakrishnan, Margaret Wrensch, Mattias Johansson, Aaron D. Norman, Alison Klein, Alyssa Clay-Gilmour, Andre Franke, Andres V. Ardisson Korat, Bill Wheeler, Björn Nilsson, Caren Smith, Chew-Kiat Heng, Ci Song, David Riadi, Elizabeth B. Claus, Eva Ellinghaus, Evgenia Ostroumova, Florent de Vathaire, Giovanni Cugliari, Giuseppe Matullo, Irene Oi-Lin Ng, James R. Cerhan, Jeanette E. Passow, Jia Nee Foo, Jiali Han, Jianjun Liu, Jill Barnholtz-Sloan, Joellen M. Schildkraut, John Maris, Joseph L. Wiemels, Kari Hemminki, Keming Yang, Lambertus A Kiemeney, Lang Wu, Laufey T Amundadottir, Marc-Henri Stern, Marie-Christine Boutron, Mark Martin Iles, Mark P. Purdue, Martin Stanulla, Melissa Bondy, Mia Gaudet, Lenha Mobuchon, Nicki J. Camp, Pak Chung Sham, Pascal Guénel, Paul Brennan, Philip R. Taylor, Puya Gharahkhani, Quinn Ostrom, Rachael Stolzenberg-Solomon, Rajkumar Dorajoo, Richard Houlston, Robert B Jenkins, Sharon Diskin, Sonja I. Berndt, Spiridon Tsavachidis, Stefan Enroth, Stephen J. Chanock, Tabitha Harrison, Tessel Galesloot, Ulf Gyllensten, Vijai Joseph, Y Shi, Wenjian Yang, Yi Lin, Stephen K. Van Den Eeden, Maria Carolina Borges, Kimberley Burrows, Rozenn N. Lemaitre, Sean Harrison, Stephen Burgess, Xuling Chang, Jason Westra, Nikhil K. Khankari, Kostas Tsilidis, Tom Gaunt, Gibran Hemani, Jie Zheng, Therese Truong, Tracy O’Mara, Amanda B. Spurdle, Matthew H. Law, Susan L. Slager, Brenda M. Birmann, Fatemeh Saberi Hosnijeh, Daniela Mariosa, Christopher I. Amos, Rayjean J. Hung, Wei Zheng, Marc J. Gunter, George Davey Smith, Caroline Relton, Richard M. Martin, Philip C. Haycock

**Affiliations:** 1 MRC Integrative Epidemiology Unit (IEU), University of Bristol, Bristol, UK; 2 Population Health Sciences, Bristol Medical School, University of Bristol, Bristol, UK; 3 Department of Medicine, University of Washington, Seattle, WA, USA; 4 MRC Biostatistics Unit, University of Cambridge, Cambridge, UK; 5 Department of Paediatrics, Yong Loo Lin School of Medicine, National University of Singapore, Singapore, Singapore; 6 Khoo Teck Puat—National University Children’s Medical Institute, National University Health System, Singapore, Singapore; 7 Department of Mathematics, Statistics, and Computer Science, Dordt College, Sioux Center, IA, USA; 8 Division of Genetic Medicine, Department of Medicine, Vanderbilt University Medical Center, Nashville, TN, USA; 9 Department of Epidemiology and Biostatistics, School of Public Health, Imperial College London, London, UK; 10 Department of Hygiene and Epidemiology, University of Ioannina School of Medicine, Ioannina, Greece; 11 Universite Paris-Saclay, UVSQ, Inserm, Gustave Roussy, Team “Exposome, Heredity, Cancer and Health”, CESP, Villejuif, France; 12 Genetics and Computational Biology Division, QIMR Berghofer Medical Research Institute, Brisbane, QLD, Australia; 13 School of Medicine, Faculty of Health Sciences, University of Queensland, Brisbane, Australia; 14 Statistical Genetics, QIMR Berghofer Medical Research Institute, Brisbane, QLD, Australia; 15 School of Biomedical Sciences, Faculty of Health, and Institute of Health and Biomedical Innovation, Queensland University of Technology, Kelvin Grove, QLD, Australia; 16 Department of Health Sciences Research, Mayo Clinic, Rochester, MN, USA; 17 Channing Division of Network Medicine, Department of Medicine, Brigham and Women’s Hospital and Harvard Medical School, Boston, MA, USA; 18 Institute for Risk Assessment Sciences, Utrecht University, Utrecht, The Netherlands; 19 Genomic Epidemiology Branch, International Agency for Research on Cancer (IARC), Lyon, France; 20 Dan L Duncan Comprehensive Cancer Center Baylor College of Medicine, Houston, USA; 21 Lunenfeld-Tanenbaum Research Institute, Sinai Health and University of Toronto, Toronto, Canada; 22 Division of Epidemiology, Department of Medicine, Vanderbilt Epidemiology Center, Vanderbilt University Medical Center, Nashville, TN, USA; 23 Section of Nutrition and Metabolism, International Agency for Research on Cancer (IARC), Lyon, France; 24 NIHR Biomedical Research Centre at University Hospitals Bristol and Weston NHS Foundation Trust and the University of Bristol, Bristol, UK

## Abstract

**Background:**

Mendelian randomization (MR) studies are susceptible to metadata errors (e.g. incorrect specification of the effect allele column) and other analytical issues that can introduce substantial bias into analyses. We developed a quality control (QC) pipe-line for the Fatty Acids in Cancer Mendelian Randomization Collaboration (FAMRC) that can be used to identify and correct for such errors.

**Methods:**

We collated summary association statistics from fatty acid and cancer genome-wide association studies (GWAS) and subjected the collated data to a comprehensive QC pipeline. We identified metadata errors through comparison of study-specific statistics to external reference data sets (the National Human Genome Research Institute-European Bioinformatics Institute GWAS catalogue and 1000 genome super populations) and other analytical issues through comparison of reported to expected genetic effect sizes. Comparisons were based on three sets of genetic variants: (i) GWAS hits for fatty acids, (ii) GWAS hits for cancer and (iii) a 1000 genomes reference set.

**Results:**

We collated summary data from 6 fatty acid and 54 cancer GWAS. Metadata errors and analytical issues with the potential to introduce substantial bias were identified in seven studies (11.6%). After resolving metadata errors and analytical issues, we created a data set of 219 842 genetic associations with 90 cancer types, generated in analyses of 566 665 cancer cases and 1 622 374 controls.

**Conclusions:**

In this large MR collaboration, 11.6% of included studies were affected by a substantial metadata error or analytical issue. By increasing the integrity of collated summary data prior to their analysis, our protocol can be used to increase the reliability of downstream MR analyses. Our pipeline is available to other researchers via the CheckSumStats package (https://github.com/MRCIEU/CheckSumStats).

## Background

Summary data from genome-wide association studies (GWAS) provide a rich resource for two-sample Mendelian randomization (MR) studies of exposure–disease pathways (see Box 1 for a general overview of MR).

To strengthen causal inference, MR studies evaluate the sensitivity of their results to violations of analytical or instrumental variable assumptions, such as the presence of horizontal pleiotropy, for which an increasingly broad and sophisticated range of methods are available.^1–3^ An additional, often overlooked source of bias in MR studies are errors in the underlying summary data or metadata. For example, incorrect specification of the effect allele column may lead to effect estimates that are in the wrong direction.^4^ These errors occur because conventions for the inclusion or naming of data fields that avoid ambiguity have not been widely adopted by the GWAS community, increasing the potential for misinterpretation by data analysts.^5^ GWAS summary data can also be obtained from an increasingly diverse range of sources, including online platforms and study-specific websites, but it is not always clear whether such results have been through post-GWAS filtering steps [e.g. with low frequency or poorly imputed single-nucleotide polymorphisms (SNPs) excluded], which increases the potential for unreliable genetic associations. The potential for metadata and summary-data errors is compounded in relatively complex MR study designs, such as in MR-PheWAS^6–8^ (MR-phenome-wide association study), wide-angled MR^7,9^ and pan-disease MR,^10^ in which summary-data sets from many different studies, corresponding to many different exposures and/or outcomes, are collated and harmonized into a single analysis.Within the GWAS field, quality control (QC) procedures have been developed that can detect a wide range of analytical issues and metadata errors, either at the GWAS stage^11^ or at the post-GWAS meta-analysis stage.^12^ For example, it is common practice to exclude genetic variants of low genotype or imputation quality or with low minor allele counts, since inclusion of such variants can lead to unstable genetic effect estimates and increase the rate of type I errors.^12^ A widely used QC strategy for the identification of metadata and summary-data errors in GWAS meta-analyses is to compare study-specific statistics to external reference data sets or to results based on theoretical expectations.^12^ Some of these QC procedures can also be used in the MR context to identify potential issues with the summary data. For example, effect allele coding errors can be identified by comparing reported allele frequency with allele frequency in a reference population. However, MR studies are subject to a unique set of challenges that often hamper the application of some previously developed QC checks. For example, to reduce the risk of individual re-identification, some consortia do not share allele frequency information with external researchers or replace it with the allele frequency of a reference population. A further hindrance is that metrics of genotype or imputation quality, or of between-study heterogeneity (in the meta-analysis context), are often not made available in GWAS results files. Some QC procedures flag potential issues by comparing study-specific statistics across studies^12^ but under the assumption that all studies employed the same regression models with the same outcomes, covariates and trait transformations, which is unlikely in complex MR study designs. Some studies only make small subsets of GWAS summary data available to researchers, which makes detecting errors harder.

In the present paper, we describe a pipeline for the QC of GWAS summary data developed for the Fatty Acids in Cancer Mendelian Randomization Collaboration (FAMRC)—a pan-cancer MR study that seeks to evaluate the causal relevance of fatty acids for risk for most major cancers. The basic principle of our QC approach is to identify metadata errors through comparison of study-specific statistics to external reference data sets [e.g. the National Human Genome Research Institute-European Bioinformatics Institute (NHGRI-EBI) GWAS catalogue and 1000 genome super populations] and to identify potential analytical issues or summary-data errors through comparison of reported to expected genetic effect sizes. Using the pipeline, we created a data set of 219 842 genetic associations with 90 cancer types, generated in analyses of 566 665 cancer cases and 1 622 374 controls in 51 studies. The size and complexity of the FAMRC make it an ideal collaboration in which to develop and evaluate QC processes for the detection of errors that can introduce biases into downstream MR analyses.

## Methods

**Figure 1:**
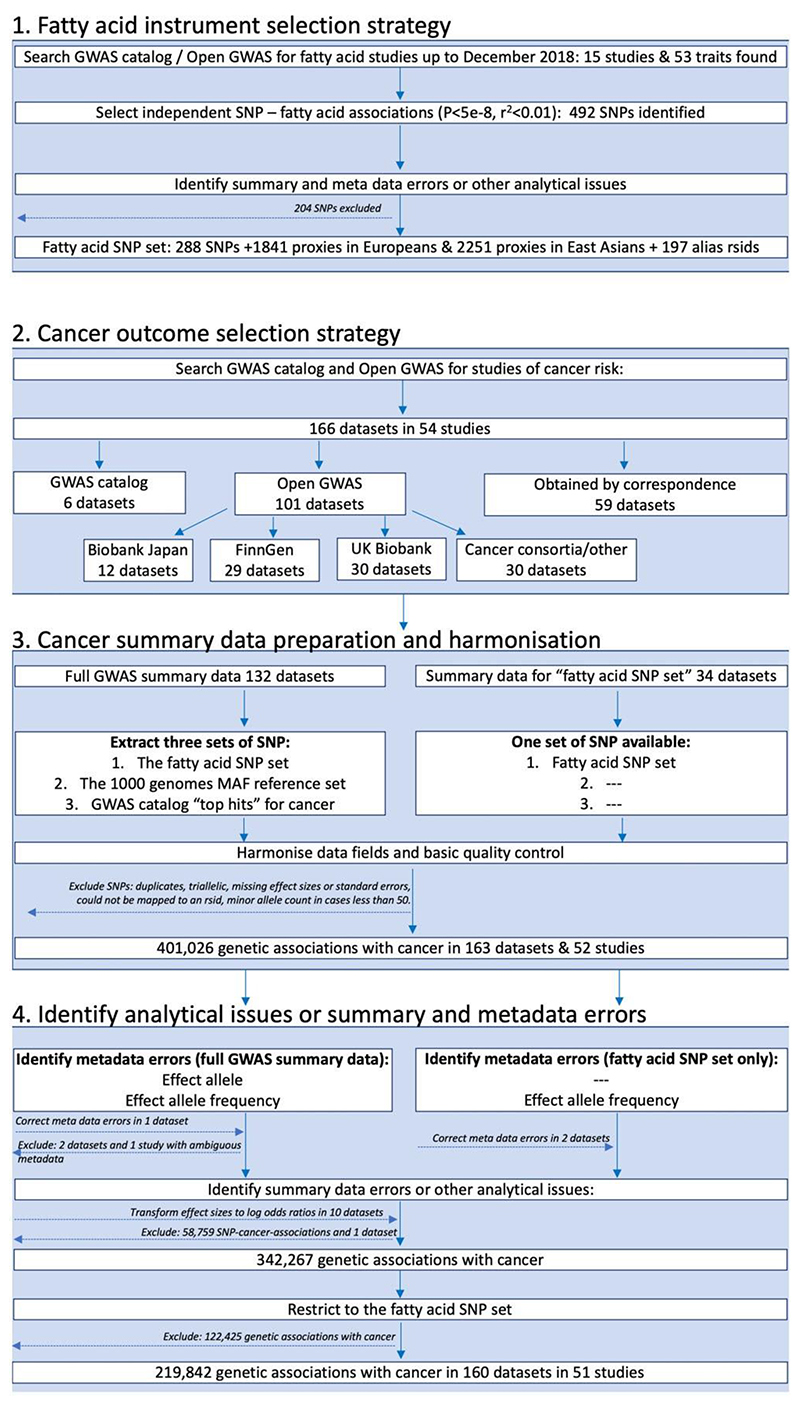
Study design flow chart. GWAS, genome-wide association study; SNP, single-nucleotide polymorphism; rsid, reference SNP identifier

The FAMRC had four key design components: (i) fatty acid instrument selection strategy; (ii) cancer outcome selection strategy; (iii) cancer data preparation and harmonization; and (iv) identification of summary-data errors, metadata errors and other analytical issues (Figure 1).

### Fatty acid instrument selection strategy

We searched for GWAS of fatty acids published up to December 2018 by searching the NHGRI-EBI GWAS catalogue^13^ (https://www.ebi.ac.uk/gwas/) and Open GWAS^14^ (https://gwas.mrcieu.ac.uk/), using fatty acid-related search terms, including: fat, acid, fatty acid, DHA, omega, mono-unsaturated, monounsaturated, polyunsaturated, saturated, omega 3 and omega 6. Fifteen studies were identified by this strategy. When full summary association statistics were available, independent genetic associations with *P* < 5e-8 were identified through linkage disequilibrium (LD) clumping (*r*^2^ threshold set to 0.01), with LD reference panels based on either the European or East Asian 1000 genome superpopulations (clumping was performed using the ieugwasr package^15^). We also selected all SNP associations reported in the GWAS catalogue, with no specified *P*-value threshold. We further identified SNP proxies, defined as SNPs having an *r*^2^ of 2:0.8 with any one of the fatty acid SNPs in European or East Asian 1000 genomes reference data. We also searched for alias reference SNP identifiers (rsids) in the Single Nucleotide Polymorphism Database (dbSNP) and 1000 genomes reference data, to make allowance for different rsids across different genome builds for the same SNP. We refer to the genetic associations for fatty acids, their *r*^2^ proxies and alias rsids as the ‘fatty acid SNP set’ (Figure 1). To identify metadata errors, summary-data errors or other analytical issues, we developed and applied a QC pipeline to the fatty acid summary-data sets (described below).

### Cancer outcome selection strategy

We searched for studies of cancer in the GWAS catalogue^13^ up to 1 November 2018. Search terms included: cancer, carcinoma, neoplasm, neoplastic, tumor, tumour, adenocarcinoma, glioblastoma, leukemia, lymphoma, melanoma, meningioma, mesothelioma, myeloma, neuroblastoma and sarcoma. When multiple studies of the same cancer outcome were identified, we prioritized the larger study. When not already available via Open GWAS^14^ (https://gwas.mrcieu.ac.uk/) or the GWAS catalogue, we invited the identified studies to share summary data for all SNPs in their GWAS analysis (defined as ‘full GWAS summary data’). If studies were unable to share full summary data, they were invited to share genetic association results for the ‘fatty acid SNP set’. We further downloaded summary association statistics for cancers from Biobank Japan^16–18^ (http://jenger.riken.jp/en/), FinnGen [data freeze 1 (14 January 2020)] (https://www.finngen.fi/fi) and UK Biobank^19,20^ using the Open GWAS platform^14^ and ieugwasr package.^15^ We prioritized studies of cancer incidence and excluded studies of cancer survival, mortality or progression-related phenotypes.

For data sets obtained via correspondence, studies were invited to share summary data up until December 2019, after which data collection was closed. Example data sharing instructions can be found in the Supplementary materials. For each SNP, we asked studies to provide a minimum of: effect estimates (log odds ratios and standard errors), the effect allele, non-effect allele and effect allele frequency. We also asked studies to provide metrics of SNP genotype quality, such as *P*-values for Hardy–Weinberg equilibrium (HWE) and metrics of imputation quality, such as info scores. When the GWAS was a meta-analysis of multiple independent studies, we additionally requested *P*-values for between-study heterogeneity.

### Cancer data preparation and harmonization

For each cancer with full summary data, we extracted the following three sets of SNPs:

- the fatty acid SNP set;
- the 1000 genomes reference set;
- the GWAS catalogue ‘top hits’ for cancer.

‘Top hits’ refers to the strongest or statistically significant genetic associations for the cancer of interest from published studies (often defined as *P*-values <5x10^−8^). When full summary data were not provided, QC analyses were restricted to the ‘fatty acid SNP set’. We next formatted the cancer summary-data sets to have similar tabular formats (e.g. where results were distributed across multiple files we merged these together) and to have consistently named data fields. SNPs without rsids were mapped to an rsid using the reported chromosome and base pair position. We excluded duplicate and triallelic SNPs as well as SNPs with missing effect sizes and standard errors or with a minor allele count of <50 in either cases or controls. If standard errors were not reported, we attempted to infer these from confidence intervals or *P*-values before excluding the SNP. We asked each study to confirm the identity of the effect allele and effect allele frequency columns in their data sets, unless this was unambiguously specified in the metadata or associated readme file. We manually mapped the cancer name for each data set to the experimental factor ontology (EFO).^21^

### QC pipeline to identify analytical issues or summary and metadata errors

**Figure 2:**
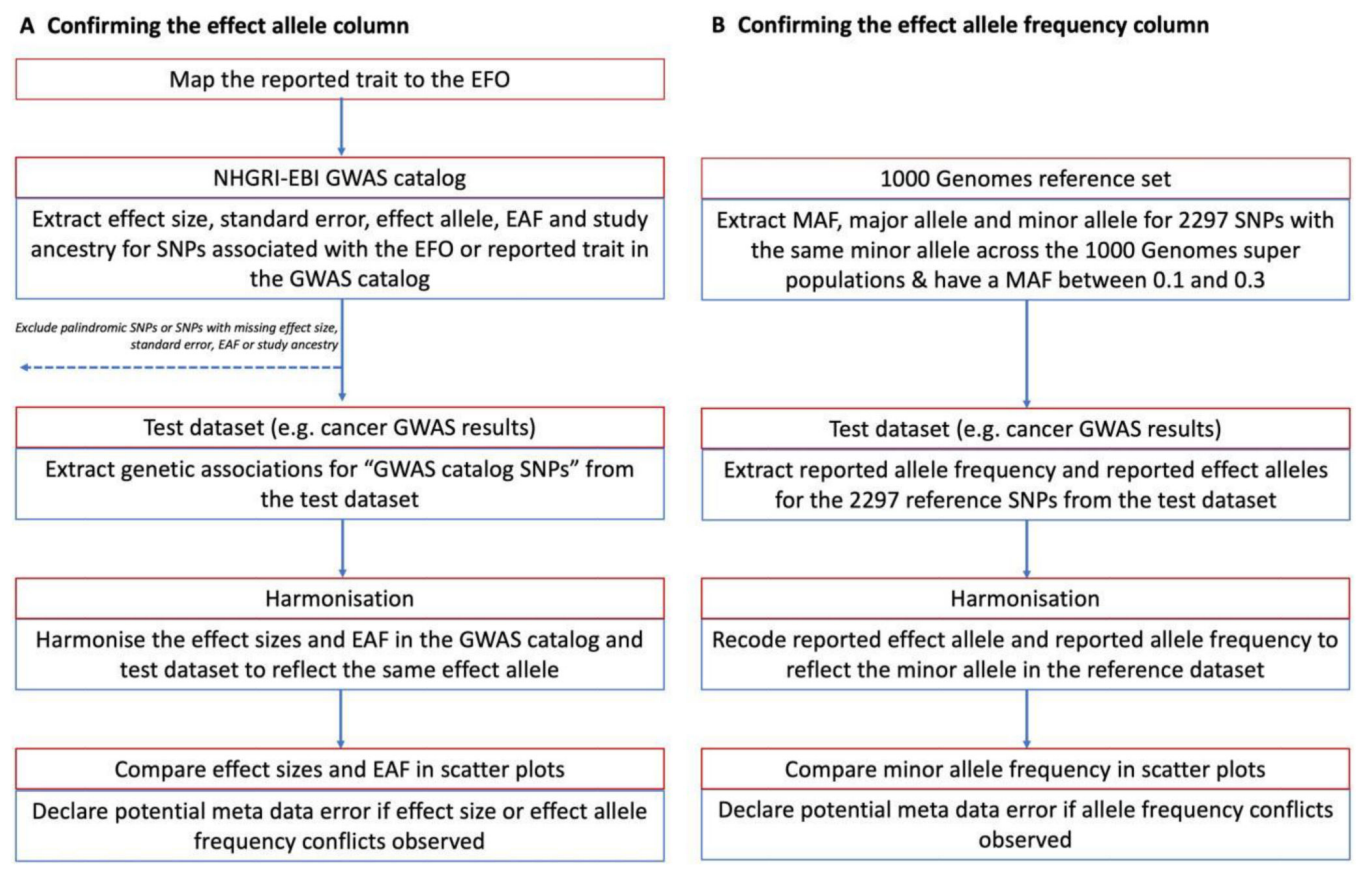
Recommended procedure to confirm the identity of the effect allele and effect allele frequency columns in the results of a genome-wide association study. EBI, European Bioinformatics Institute; EFO, experimental factor ontology; EAF, effect allele frequency; GWAS, genome-wide association study; MAF, minor allele frequency; NHGRI, National Human Genome Research Institute; SNP, single-nucleotide polymorphism

To identify metadata errors, summary-data errors or other analytical issues, we developed a QC pipeline based on the R programming language and associated packages.^15,22–32^ We used the pipeline to: (i) confirm the identity of the effect allele column (Figure 2); (ii) confirm the identity of the effect allele frequency column (Figure 2); and (iii) identify analytical issues or potential errors in the summary data (e.g. an unusual number of GWAS hits or unusual distributions in effect sizes). All the functions and tests of the QC pipeline are available to other researchers via the CheckSumStats package (https://github.com/MRCIEU/CheckSumStats).

### Instrument-specific QC

To identify potential analytical issues or errors in the genetic instruments for fatty acids, we compared genetic association results identified through LD clumping (*r*^2^¼0.01 and kb ¼ 10 000) to associations in the GWAS catalogue. We set the significance threshold for LD clumping to the threshold reported in the fatty acid GWAS: 5-e8 in CHARGE (Cohorts for Heart and Aging Research in Genomic Epidemiology Consortium),^33^ SCHS (the Singapore Chinese Health Study)^34^ and NHAPC/MESA-CHI (Nutrition and Health of Aging Population in China/Multi-Ethnic Study of Atherosclerosis—Chinese ancestry participants),^35^ 1e-8 in the FHS (the Framingham study),^36^ 2.3e-9 in Kettunen *et al*.^37^ and 1.03e-10 in the TwinsUK/KORA (Twins United Kingdom/Cooperative Health Research in the Region of Augsburg) study.^38^ We used the 1000 genomes European superpopulation as an LD reference panel for CHARGE, the FHS, Kettunen *et al*. and TwinsUK/KORA, and the East Asian superpopulation as an LD reference panel for the SCHS and NHAPC/MESA. We searched the GWAS catalogue for the lead SNP, identified by the latter clumping procedure, as well as SNPs within 200 000 base pairs of the lead SNP (associations were retrieved from the GWAS catalogue via the gwasra-pidd package^32^). Data sets were flagged for further investigation if any lead SNPs were absent from the GWAS catalogue. We additionally searched for metadata and summary-data errors in the fatty acid GWAS results through comparisons of effect alleles and allele frequency with external reference data sets and by comparing reported to expected effect sizes (described below).

### Confirming the effect allele column

To identify incorrect specification of the effect allele column, we compared summary association statistics in the test data set (either a fatty acids or cancer data set) to summary association statistics in the NHGRI-EBI GWAS catalogue^13^ (Figure 2). The latter is a manually curated database of 251 401 genetic associations from 4961 publications (as of April 2021) and includes information on effect alleles, effect sizes and EFOs. The genetic associations in the manually curated database typically correspond to the statistically significant findings (‘top hits’) from published studies (often defined as *P* < 5e-8). In recent years, the GWAS catalogue has started to host full GWAS summary statistics. However, for this QC step, we are referring exclusively to the manually curated database of published ‘top hits’.

In the first step, we searched the GWAS catalogue for SNPs associated with the EFO or reported trait of the test data set. Second, for each SNP associated with the EFO term, we extracted from the GWAS catalogue the effect size, standard error, effect allele, effect allele frequency and study ancestry (genetic associations were retrieved via the gwasrapidd package^32^). SNPs missing any of this information, or that were palindromic, were removed. Third, genetic associations for these SNPs were then extracted from the test data set. Fourth, the effect sizes and effect allele frequencies from the GWAS catalogue and test data set were harmonized to reflect the same effect allele and compared in scatter plots (constructed using the ggplot package^22^). Comparisons were restricted to populations of European or East Asian ancestry.

We then inspected the scatter plots for conflicting directions of association. For example, we declared a conflicting direction of association if the effect allele was associated with higher cancer risk in the GWAS catalogue but was associated with lower risk in the test data set. The level of conflict was further labelled as ‘high’ if the *P*-value for the association was <0.0001 in both the GWAS catalogue and the test data set and as ‘moderate’ if not, so as to make allowance for chance deviations in effect direction in small studies. If the test data set and the data in the GWAS catalogue corresponded to the same publication, the conflict level was labelled as ‘high’ regardless of the *P*-value strength. For comparisons of allele frequency, we declared a conflict if the effect allele frequency was not greater (or less) than 0.5 in both data sets. The level of conflict was further labelled as high if the minor allele frequency was S0.4 in both data sets, and as moderate if not. The latter step makes allowance for chance deviations in allele frequencies for SNPs with minor allele frequencies close to 0.5. Conflicts were also labelled as high if the allele frequency differed by >10 points between the test and reference data sets. When interpreting the scatter plots, it is important to take into account the total number of SNPs in the comparison as well as the ancestry of the test and reference data sets. Conflicting associations are more likely to reflect true effect allele coding issues when the conflict is systematic across a large number of SNPs and when the ancestry of the data sets being compared is the same. When a substantial proportion of SNPs displayed effect or allele frequency conflicts, we flagged the test data set as containing a potential effect allele metadata error.

### Confirming the effect allele frequency column

To confirm the effect allele frequency column, we compare the test data sets to two types of reference data sets: the 1000 genomes project^39^ and the exposure study. In the case of the present analysis, we used CHARGE and the SCHS as representative of exposure (i.e. fatty acid) studies in Europeans and East Asians, respectively. For comparisons with the 1000 genomes project, we created a reference data set of 2297 SNPs that have the same minor allele across the African, European, East Asian, American, South Asian and Global super populations and that also have a minor allele frequency of between 0.1 and 0.35 (this data set is available to other researchers in the CheckSumStats R package) (Figure 2). We refer to the 2297 SNPs as the ‘1000 genomes reference set’. For comparisons with CHARGE and the SCHS, we created a reference data set corresponding to the fatty acid SNP set described above (see ‘Fatty acid instrument selection strategy’). We then compare minor allele frequencies between the test data set and the reference data set. The comparison involves the following steps. First, we merge the test and reference data set. Second, we recode the reported effect allele and reported effect allele frequency in the test data set to reflect the minor allele in the reference data set. Third, we compare minor allele frequencies between the data sets in scatter plots^22^ and inspect the plots for conflicting patterns. A conflict is declared for individual SNPs if their allele frequency is >0.5 in the test data set. If the frequency is also 2:0.58, the conflict level is upgraded to ‘high’ (to make allowance for chance deviations). Conflicts are also labelled as high if the allele frequency differs by >10 points between the test and reference data sets. If an inverse correlation is observed across the vast majority of SNPs, this indicates that the conflict is systematic and that the reported effect allele frequency actually corresponds to the non-effect allele. When there is a conflict for approximately half the SNPs, this implies that the reported effect allele frequency column actually corresponds to the minor allele and that the minor allele is not consistently the effect allele. In the latter situation, the scatter plot will show two separate groups of SNPs—one with a positive correlation and the other with an inverse correlation—in the allele frequency between the data sets. The strength and linearity of the correlation in the allele frequency between the test and reference data sets also provide information on the ancestral background of the participants used to generate the test data set. An advantage of using our ‘1000 genomes reference set’ is that incorrect specification of the effect allele frequency can be identified without knowledge of the ancestral background of the test data set.

### Identifying other analytical issues and summary-data errors

To identify potential analytical issues or summary-data errors, we compare the expected and reported effect sizes. For continuous exposures, such as fatty acid levels, we generate expected betas using the formula:

$$\;beta\;=\frac{z}{\sqrt{2p(1-p)\left(n+z^{2}\right)}}$$

where *p* is the minor allele frequency, *n* is the sample size and *z* is the ratio of the effect size to its standard error. The predicted effect size from this transformation can be interpreted as the standard deviation change in the exposure per copy of the effect allele (assuming that *z* itself was generated in an additive genetic model). When the expected effect size is a log odds ratio, e.g. for cancer status analysed in a logistic regression model, we generate the expected log odds ratio for each SNP using a simulation method that takes into account the SNP’s z-score, minor allele frequency and the number of cases and controls^40^ and assumes an additive genetic model. More details of the method can be found in the Supplementary materials.

We then regress the expected effect size (the per-allele standard deviation change in a continuous trait or log odds ratio for a binary trait) on the reported effect size and interpret a slope very different from 1 (which we define as either >1.20 or <0.8) as evidence for an unusual distribution in the reported effect sizes. We also assess the overall shape of the relationship between the expected and reported effect sizes in scatter plots, with the expectation of linearity. Deviations of the slope from one or non-linear patterns could reflect:

- errors in the reported effect sizes, reported sample sizes or reported allele frequencies;
- effect size scale conflicts [e.g. reported effect sizes have not been standardized (for continuous traits) or effect sizes have not been generated in a logistic regression model (for binary cancer outcomes)]);
- the impact of covariate adjustment in the regression model;
- deviations from HWE.

If we found that the summary association statistics for cancer were generated in a linear model (e.g. BOLT-LMM^41^), we transformed the effect size to a log odds ratio scale using the following formula:

$$\log\;odds\;=\frac{\;beta\;}{u(1-u)}$$

where *u* is the case prevalence in UK Biobank. The standard error for the log odds ratio can be obtained with the same transformation.

To see whether discrepancies between the reported and expected effect sizes were related to metrics of genotype or imputation quality, we compared discrepancies to reported info or *r*^2^ imputation scores, *P*-values for HWE and, in the case of meta-analyses, *P*-values for between-study heterogeneity and the number of studies. Potential errors in reported effect sizes were also identified by comparing *z_b_*-scores (inferred from the reported effect size and standard error) to *z_p_*-scores (inferred from the reported *P*-value) in scatter plots (also known as P–Z plots^12^).

## Results

### Fatty acid data sets

Our search of the GWAS catalogue identified 15 publications corresponding to 71 fatty acid traits, including 13 monounsaturated fatty acids (MUFAs), 22 saturated fatty acids, 6 omega 3 polyunsaturated fatty acids (PUFAs), 8 omega six PUFAs, 8 trans-fatty acids and 14 other fatty acid characteristics (Supplementary Tables S1 and S2, and Supplementary Figure S1).^33–38,42–50^ The median number of fatty acids assessed per publication was 6 (minimum ¼ 2, maximum ¼ 34). Nine of the 15 publications were conducted by, or overlapped with, the CHARGE consortium. The median study sample size was 7811 (minimum ¼ 284; maximum ¼ 17 267). The 15 publications corresponded to seven independent studies or consortia. An interaction study was the only fatty acid GWAS excluded.^51^ We subsequently invited the identified studies to share full summary data with the FAMRC (except for Shin *et al*.^38^ and Kettunen *et al*.,^52^ which were already available via Open GWAS^49^). The vast majority of the GWAS analyses were conducted in European ancestry populations (11/15), two were conducted in populations of East Asian ancestry, one in a population of South Asian ancestry and one in a transethnic GWAS of Europeans and East Asians. We collated full summary data from 13 of 15 publications, corresponding to six independent consortia or cohort studies: CHARGE,^33,42–44,47–50^ SCHS,^34^ FHS,^36^ the TwinsUK/KORA study,^38^ the NHAPC/MESA-CHI study^35,47^ and Kettunen *et al*.^37^ In the SCHS, fatty acid GWAS analyses were conducted separately amongst myocardial infarction cases and controls. We combined these data sets by fixed-effects meta-analysis in METAL.^53^ Overall, 124 summary-data sets were available from the six studies (where each data set corresponds to a single fatty acid GWAS analysis, Supplementary Table S2).

**Figure 3:**
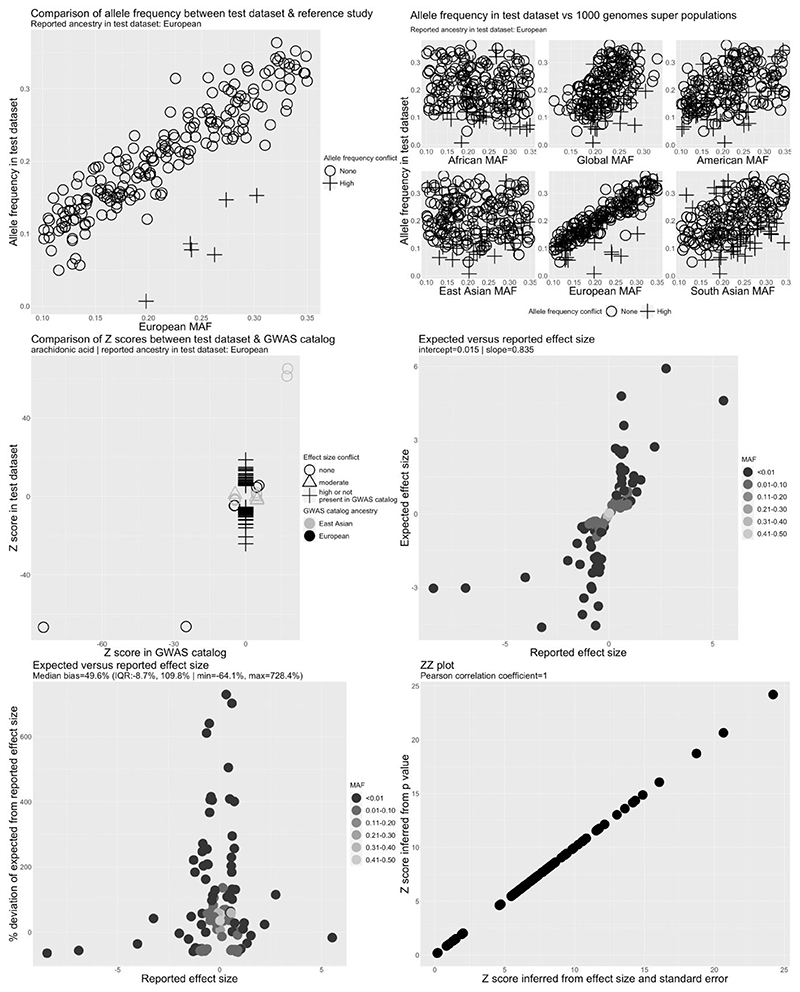
Quality control report for genetic summary data from a genome-wide association of arachidonic acid in the Cohorts for Heart and Aging Research in Genomic Epidemiology Consortium (CHARGE). Allele frequencies are expected to be <0.50. A high allele frequency conflict is defined as an allele frequency of >0.58 in the test data set (CHARGE in this example) or if the allele frequency differs by >10 points between the test and reference data sets. Moderate allele frequency conflicts are allele frequencies of >0.50 but S0.58. Effect size conflicts are defined as different directions of effect, represented by signed z-scores, between the test data set (CHARGE in this example) and the GWAS catalogue. The level of conflict is further labelled as ‘high’ if the *P*-value for the association is <0.0001 in both the GWAS catalogue and the test data set, and as ‘moderate’ if not. Effect allele frequency conflicts with the GWAS catalogue are declared if the effect allele frequency is not greater (or less) than 0.5 in both data sets. The level of conflict is further labelled as high if the minor allele frequency is S0.4 in both data sets, and as moderate if not. Effect allele frequency conflicts are also defined as high if the effect allele frequency differs by >10 points between the test and reference data sets. EAF, effect allele frequency; GWAS, genome-wide association study; MAF, minor allele frequency

To identify metadata and summary-data errors, we applied a custom QC pipeline to the CHARGE, FHS, SCHS, TwinsUK/KORA, NHAPC/MESA-CHI and Kettunen *et al*. studies (Figure 3 and Supplementary Figures S1–S6). No allele frequency or effect allele conflicts were observed, indicating that the reported effect allele and effect allele frequency columns were correctly indicated. A strong and positive linear relationship between the expected and reported effect sizes was also observed in the FHS, TwinsUK/KORA, NHAPC/MESA-CHI and Kettunen *et al*. studies, with slopes close to 1, suggesting the absence of major analytical issues in these studies.

The expected and reported effect sizes for selected fatty acids were not, however, well correlated in the CHARGE (Figure 3) and SCHS studies (Supplementary Figure S5). We also identified 109 independent GWAS hits for arachidonic acid in CHARGE after LD clumping, of which only four were also reported in the GWAS catalogue, compatible with the presence of a large number of false positives. After corresponding with the data provider, we were able to confirm that post-GWAS filtering of low-quality variants (defined as SNPs with minor allele frequency of <5%, imputation *r*^2^ < 0.5 or as SNPs that were present in only one study^33^) had not been performed on the data set posted to the CHARGE website. After excluding these SNPs, following recommendations of the data provider,^33^ we observed a strong linear relationship between the reported and expected effect sizes and a slope of 1.02 (Supplementary Figure S7). The 109 independent GWAS hits also decreased to seven in the cleaned data set, all of which mapped to the fatty acid desaturase genomic region on chromosome 11 or Pyridoxal Dependent Decarboxylase Domain Containing 1 (*PDXDC1*) on chromosome 16, regions harbouring established GWAS hits for fatty acids (and therefore unlikely to be false positives). In the SCHS, the relationship between the expected and reported effect sizes was skewed by a single outlier SNP (Supplementary Figure S5). Further investigation revealed that the outlier was due to incorrect specification of the sample size for this SNP.

We also identified two independent GWAS hits for selected fatty acids in the NHAPC/MESA-CHI and SCHS studies that were not present in the GWAS catalogue or in the associated publications. We subsequently confirmed that post-GWAS filtering steps for low-quality variants had not been applied to the GWAS results files for the NHAPC/MESA-CHI study and that the identified GWAS hit had failed the reported QC checks (we therefore excluded this variant). In the SCHS, correspondence with the data provider indicated that a file sharing error had occurred and we therefore obtained a new set of GWAS results files (in which conflicts with the GWAS catalogue were no longer observed). Conflicts with the GWAS catalogue were not observed for the FHS, TwinsUK/KORA and Kettunen *et al*. studies. The false positive GWAS hits identified in the CHARGE and NHAPC/MESA-CHI studies only apply to the results files shared with the FAMRC and do not apply to the published GWAS findings.^33–35,42–44,47–50^

After applying the SNP selection strategy and resolving the analytical issues flagged by the QC pipeline, we identified 288 SNPs associated with 53 fatty acid traits (median 6 per trait). We identified a further 1841 SNP proxies using the 1000 genomes European super population, 2251 SNP proxies in the 1000 genomes East Asian super population and 197 alias rsids in dbSNP and 1000 genomes reference data. The total number of SNPs associated with fatty acids, their *r*^2^ proxies and alias rsids was 2326 for European studies and 2596 in East Asians (excluding duplicate SNPs that overlapped amongst the fatty acid and proxy sets) (Supplementary Tables S3 and S4). We henceforth refer to these SNPs as the ‘fatty acid SNP set’.

### Collation of cancer data sets

As of January 2020, we had collated 166 summary genetic data sets from 54 cancer studies^16,54–102^ (Supplementary Table S5 and Supplementary Figure S8). Thirty-eight studies supplied a single data set, 10 studies supplied two to five data sets and 6 studies supplied more than five data sets. Of the 166 cancer data sets, 31 were from UK Biobank, 12 were from Biobank Japan and 29 were from FinnGen. Fifty-nine data sets (from 41 studies) were obtained via correspondence with study principal investigators, 6 data sets (from 3 studies) were downloaded from the GWAS catalogue and 101 data sets (from 12 studies) were downloaded from the Open GWAS project.^14^ Of the 101 Open GWAS data sets, 12 were from Biobank Japan, 29 were from FinnGen, 30 were from UK Biobank, 27 were from GWAS meta-analysis consortia and 3 were from other studies. Further details of the cancer studies can be found in Table 1 and \Supplementary Tables S5 and S6. Effect allele frequency was available in 156 data sets (from 46 studies), metrics of imputation quality (*r*^2^ or info scores) were available in 53 data sets (from 25 studies) and *P*-values for deviations from HWE were available in 6 (from 6 studies). Of 65 data sets derived from 29 GWAS meta-analyses, *P*-values for between-study heterogeneity were available in 18 (from 9 meta-analyses) and the number of studies per SNP was available in 16 (from 7 meta-analyses).

We extracted three sets of genetic associations from each data set for which full GWAS results were available (132 data sets from 31 studies): (i) the fatty acid SNP set, (ii) the 1000 genomes reference set and (iii) known cancer hits in the GWAS catalogue. For 34 data sets from 25 studies, only a subset of GWAS results, corresponding to the fatty acid SNP set, was available. We excluded duplicate and triallelic SNPs; SNPs with missing effect allele, effect sizes or standard errors; SNPs that could not be mapped to an rsid; and SNPs with a minor allele count in cases of <50. After these exclusions, there were 401 026 genetic associations with cancer across 163 data sets in 52 studies. Of these, 223 970 genetic associations corresponded to the fatty acid SNP set, 93 121 corresponded to the 1000 genomes reference set and 24 860 corresponded to known cancer associations in the GWAS catalogue. Three studies providing genetic associations for the fatty acid SNP set provided an additional 40 582 genetic associations for SNPs within 500 kb of a fatty acid index SNP [ACCC (ID3), UCSF_AGS þ SFAGS (ID133) and UCSF_Mayo (ID 134)] (cancer study abbreviations explained in Supplementary Table S6).

### Results of QC pipeline applied to the cancer data sets

**Figure 4:**
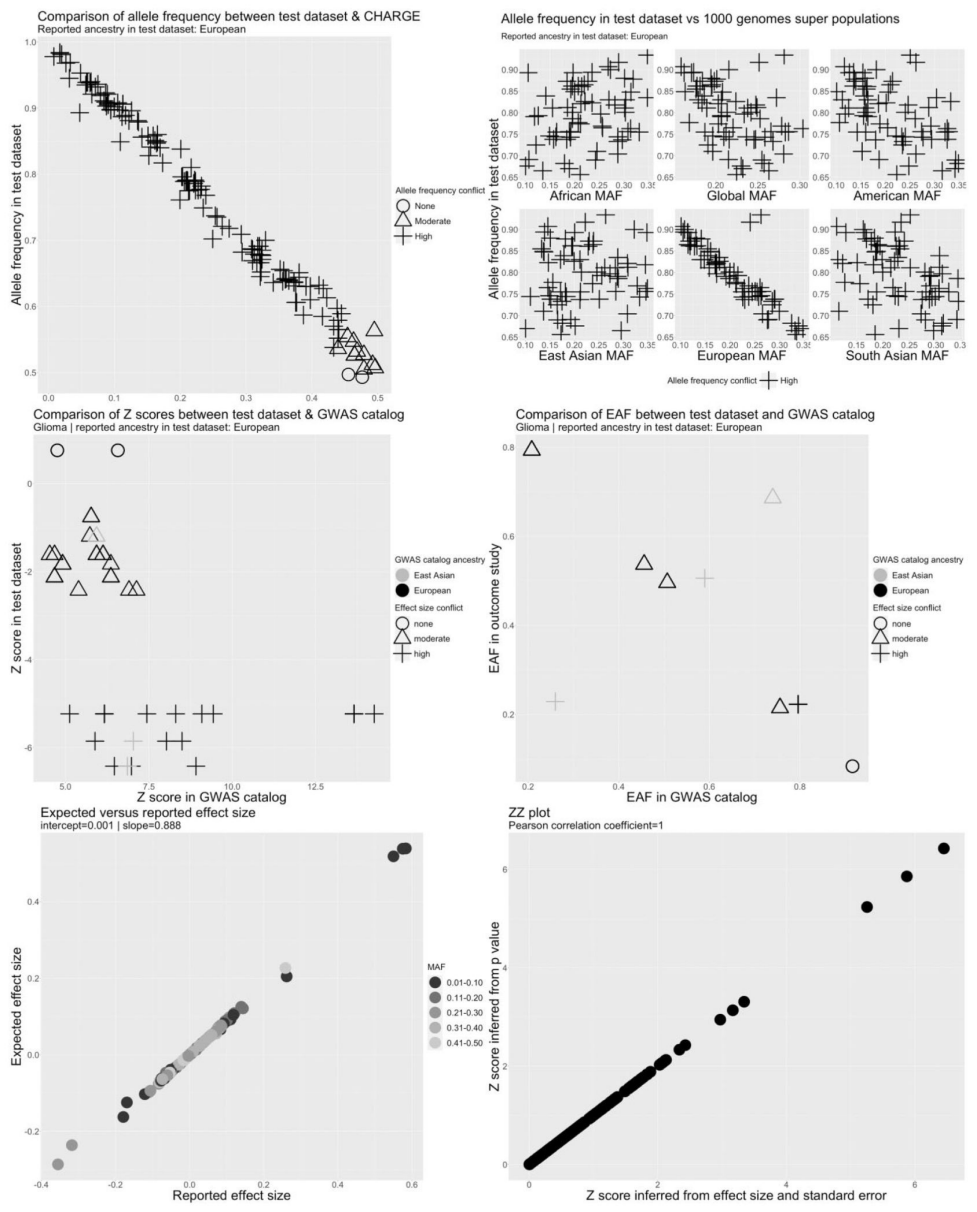
Quality control report for genetic summary data from a genome-wide association of glioma in the GliomaScan data set (ID 967). Allele frequencies are expected to be <0.50. A high allele frequency conflict is defined as an allele frequency of >0.58 in the test data set (GliomaScan in this example) or if the allele frequency differs by >10 points between the test and reference data sets. Moderate allele frequency conflicts are allele frequencies of >0.50 but S0.58. Effect size conflicts are defined as different directions of effect, represented by signed z-scores, between the test data set (GliomaScan in this example) and the GWAS catalogue. The level of conflict is further labelled as ‘high’ if the *P*-value for the association is <0.0001 in both the GWAS catalogue and the test data set, and as ‘moderate’ if not. Effect allele frequency conflicts with the GWAS catalogue are declared if the effect allele frequency is not greater (or less) than 0.5 in both data sets. The level of conflict is further labelled as high if the minor allele frequency is S0.4 in both data sets, and as moderate if not. Effect allele frequency conflicts are also defined as high if the effect allele frequency differs by >10 points between the test and reference data sets. CHARGE, Cohorts for Heart and Aging Research in Genomic Epidemiology Consortium; EAF, effect allele frequency; GWAS, genome-wide association study; MAF, minor allele frequency

Metadata errors or analytical issues were identified in 41 cancer data sets from 20 studies (Supplementary Table S7). These included serious metadata errors (defined as incorrect labelling of the effect allele or effect allele frequency columns) in five data sets from five studies. In three data sets, alleles associated with higher cancer risk in the GWAS catalogue were associated with lower risk in the test data set for a substantial proportion of SNPs (Figure 4 and Supplementary Figures S9 and S10), suggesting that the effect allele column refers to the non-effect allele. This was clearest for GliomaScan (ID 967) where 19/21 SNPs were discordant with the GWAS catalogue but was less clear for the NBS (ID 106) and BC-NHL (ID 5) data sets (cancer study abbreviations explained in Supplementary Table S6). In the BC-NHL, although all SNPs were discordant to the GWAS catalogue, the number available for comparison was small and z-scores were not large (*z* S 2.5). Therefore, we could not rule out chance deviations from the GWAS catalogue for this data set. In addition, the ancestry of the BC-NHL (East Asian) was different to the ancestry of the reference data set (European). Therefore, the observed conflict for the BC-NHL data set could also reflect differences in LD between populations. In the NBS (ID 106), equal numbers of SNPs were highly discordant and highly concordant to the GWAS catalogue. Due to the ambiguity of the effect allele we decided to drop the BC-NHL (ID 5) and NBS (ID 106) data sets. Reported effect alleles were compatible with reported cancer hits in the GWAS catalogue for other cancer data sets (Supplementary Figure S13).

We identified three data sets in which reported allele frequency was inconsistent with allele frequency in reference data sets (Figures 4 and Supplementary Figures S11 and S12). This included GliomaScan (ID 967) in which the allele frequency was inversely correlated with the allele frequency across all SNPs in ancestry matched reference data sets, indicating that the reported effect allele frequency corresponded to the non-effect allele (Figure 4). In the UCSF_AGS/SFAGS (ID 133) and TNC (ID ¼ 132), there were two groups of SNPs showing positive or inverse correlations with the allele frequency in ancestry matched data sets (Supplementary Figures S11 and S12; cancer study abbreviations explained in Supplementary Table S6), indicating that the reported effect allele frequency actually corresponds to the minor allele frequency and that the minor allele was not consistently the effect allele. For these data sets, we decided to set the effect allele frequency to missing. Allele frequency conflicts were not observed for other cancer data sets (Supplementary Figures S14–S17).

We identified 35 data sets (from 15 studies) in which the reported effect sizes had an unusual distribution when compared with expected log odds ratios, suggesting potential summary-data errors or analytical issues (Supplementary Figure S18). Further investigation revealed that summary data for 10 of the 35 data sets had been generated in linear mixed models of cancer in UK Biobank. Effect sizes from such models can be interpreted as the change in absolute cancer risk per copy of the effect allele. We retained these data sets but transformed the reported effect size into a log odds ratio scale.

Of the remaining 25 data sets (from 14 studies), we confirmed that the reported effect sizes were log odds ratios by consulting the original study publications. For 18 data sets (from nine studies), the discrepancy between reported and expected effect sizes was largely attributable to incorrect sample sizes, low imputation quality or a small number of outlier SNPs with unusually large log odds ratios (e.g. log odds ratios >1 or < –1). For example, in eight data sets from the ACCC, GICC/MDA, GECCO and HNMSC studies, we incorrectly assumed that the number of participants contributing to analyses was constant across SNPs (Supplementary Figures S19–S22; cancer study abbreviations explained in Supplementary Table S6). This inconsistency might introduce bias into methods that assume a constant sample size across SNPs (e.g. methods that make use of external LD reference panels; see ‘Discussion’).

In three data sets from the TNC, NB-UGC and ECAC studies, the discrepancy between the reported and expected effect sizes was partly attributable to low imputation quality for some SNPs (Supplementary Figure S23; cancer study abbreviations explained in Supplementary Table S6). We also found that the percentage deviation of the expected from the reported log odds ratio was strongly and inversely related to metrics of imputation quality (Supplementary Figure S24) but not *P*-values for deviation from HWE or *P*-values for heterogeneity between studies (Supplementary Figures S25 and S26). In 53 data sets (from 25 studies) in which information on imputation quality was available, there were 46 534 SNPs with imputation quality scores of <0.8, including 1119 in the fatty acid SNP set.

Discrepancies between the reported and expected effect sizes in seven data sets from OCAC and PRACTICAL were mainly attributable to a small number of SNPs with unusually large log odds ratios (>1 or < –1) (Supplementary Figure S18; cancer study abbreviations explained in Supplementary Table S6). The number of SNPs across all data sets with log odds ratios of >1 or < –1 was 368, including five SNPs in the fatty acid SNP set. Additional potential problems in reported effect sizes were identified in two data sets from the UCSF_AGS/SFAGS and ILCCO studies, where the correlation was <0.99 between zp-scores (z-scores inferred from P-values) and zb-scores (z-scores inferred from reported effect sizes and standard errors) (Supplementary Figures S11 and S27,). In one data set, this was due to three SNPs with very large effect sizes (z > 99) but with P-values very close to 1 (>0.9). The second data set showed a very irregular non-linear relationship between the two sets of z-scores (Supplementary Figure S27,). This data set was excluded. Correlations between the zb- and zp-scores were >0.99 across other cancer data sets (Supplementary Figure S28).

### Final collection of cancer summary-data sets

Application of the QC pipeline to cancer data sets led to the exclusion of 3 data sets and 1 study, leaving 160 data sets from 51 studies (Supplementary Figure S29). The retained cancer data sets represent 90 unique cancer types distributed cross 30 tissue or organ sites and were generated in analyses of 566 665 cancer cases and 1 622 374 controls (Supplementary Figure S30; Table 1 and Supplementary Tables S5 and S6).^16,54–95,98–102^ The median number of cases per study was 2442 (minimum ¼ 95; maximum ¼ 122 977) (Supplementary Figure S31). Fifteen studies reported >10 000 cases, 25 studies reported 1000–10 000 cases and 11 studies reported <1000 cases.

## Discussion

Our pipeline flagged analytical issues, metadata and summary-data errors in 23 studies (2 fatty acid GWAS and 21 cancer GWAS), including errors in 7 studies with the potential to introduce substantial bias into downstream MR analyses. These included a large number of false positive genetic associations for fatty acids and incorrect specification of the effect allele and effect allele frequency columns. Other more minor issues included inconsistent effect size scales amongst cancer studies, incorrect assumptions about sample sizes across SNPs and outlier SNPs with unusually large effect sizes.

### Effect allele metadata errors

Of the issues identified, incorrect specification of the effect allele column is the most serious, as it will lead to inferences of causal effect in the wrong direction^103,104^ (when the null hypothesis is false) and was flagged in 3 of 54 cancer studies. A related, albeit less serious, error is incorrect specification of the effect allele frequency column, which can cause harmonization problems for palindromic SNPs. Failure to harmonize palindromic SNPs between exposure and outcome studies may lead to increased heterogeneity in MR findings, which could in turn bias results towards the null (assuming the null hypothesis is false and that the palindromic SNPs are valid instruments). A conventional approach for avoiding these metadata errors is to compare allele frequency between the GWAS of interest and an external reference data set^12^ or to confirm the effect allele through correspondence with study authors (especially when these are ambiguously labelled) or through consultation of readme files. Despite performing the latter checks, five cancer studies were still affected by effect allele meta-data errors. One of the metadata errors was introduced by the FAMRC data analyst whereas others were potentially due to human error by data providers. Our approach of comparing summary associations statistics for known ‘top hits’ between the GWAS of interest and the GWAS catalogue offers an additional safeguard against such errors.

### False positive GWAS hits

False positive genetic associations for fatty acids were identified in two of six fatty acid consortia. Failure to account for false positive hits could lead to the inclusion of genetic variants in MR analyses that are not truly associated with the exposure [a violation of instrumental variable assumptions (see Box 1)], which could have the effect of biasing MR findings towards the null (assuming the null hypothesis is false). The false positives arose because we designed our instruments using the full summary association statistics, downloaded from the consortium website or obtained via correspondence, that had not gone through post-GWAS filtering procedures (e.g. exclusion of low frequency or low imputation quality variants). This instrument design strategy is probably more susceptible to inclusion of false positive genetic associations compared with using the manually curated findings described in a GWAS publication. The latter are subject to relatively rigorous reporting standards, whereas there is little consensus on the format that GWAS results should take when posted to study-specific websites. Online platforms and databases that aggregate full summary association statistics from different studies may also be susceptible to this kind of error.

It is important to consider the impact of sample size when interpreting the presence of GWAS hits in the test data set that are absent from the GWAS catalogue. For example, if the GWAS being investigated is unpublished and is larger than any previously published study, we can reasonably expect a number of genetic associations to be identified that are absent from the GWAS catalogue but are nevertheless true novel hits. When the GWAS being investigated is smaller than any previously published study, one should be more sceptical of any GWAS hits that are previously unreported.

### Inconsistent effect size scales

We also found that cancer studies did not consistently express effect sizes as log odds ratios, with a substantial proportion of cancer analyses within UK Biobank expressing effect sizes as absolute changes in disease risk. The cancer analyses in question employed BOLT-LMM—a linear mixed model that allows the inclusion of related individuals, is more powerful and efficient than conventional regression procedures^41^ and is a widely used method for analysing binary disease traits in large-scale biobanks.^105^ In general, failure to account for effect size scale differences will hamper comparison of findings amongst different studies and could lead to the misinterpretation of results.

### Summary-data errors

Potential summary-data errors were flagged by mismatches between expected and reported effect sizes. We found that a substantial proportion of the mismatches were attributable to imputed SNPs, SNPs with incorrect sample sizes and SNPs with unusually large effect sizes. The sample size errors were due to the strategy of using the maximum reported sample size to represent sample size across all SNPs. However, not all samples in a GWAS necessarily contribute to the analysis of every SNP, which is particularly common in large meta-analysis consortia with many independent studies. Incomplete sample overlap amongst SNPs within a GWAS could introduce bias into methods that assume a constant sample size, such as summary-data methods that rely on an external LD reference panel to model the correlation structure amongst SNPs in a genetic instrument. In the presence of incomplete sample overlap amongst SNPs, the use of an external LD reference panel could lead to the overestimation of the covariance in SNP effect sizes. For example, in the most extreme case of zero sample overlap, the correlation in effect sizes for two SNPs will be zero even if those two SNPs are in LD.^106^

### General recommendations

When obtaining summary GWAS data via correspondence with study authors, we recommend that researchers should request access to full GWAS summary data, as this allows a far more comprehensive assessment of summary-data reliability than is possible with only subsets of data. When full access is not possible, researchers should request summary data for SNPs that are established GWAS hits for their outcome of interest (i.e. not just the SNPs being used to instrument the exposure), which can then be used to confirm the identity of the effect allele through comparisons with the GWAS catalogue. In addition, researchers could request summary data corresponding to the SNPs in our 1000 genomes reference set, which contains 2297 SNPs with the same minor allele across all 1000 genomes super populations, and which can be used to identify allele frequency issues. An advantage of using our 1000 genomes reference set is that effect allele frequency conflicts can be identified without knowledge of the ancestral background of the test data set. Alternatively, a similar QC check can be achieved by comparing allele frequencies between the exposure and outcome studies of interest (assuming they are closely matched on ancestry). Where possible, researchers should also confirm the identity of the effect allele metadata through correspondence with the data providers.

We also recommend that researchers confirm with data providers the nature of all post-GWAS filtering procedures that have been applied to the summary data. For example, in our own collaboration, we ask each cancer study to confirm that their summary data have been through the same QC procedures as described in their GWAS publications. Failure to perform this check could lead to the inclusion of large numbers of low-quality and unreliable genetic associations. It is also advisable to confirm effect size scales, to support the correct interpretation of results. These considerations supplement previously developed guidelines for conducting MR studies.^4,107,108^

Our approach of comparing expected to reported effect sizes, and of comparing summary association statistics to external reference data sets, offers an additional safeguard against the aforementioned errors and analytical issues. A limitation of this approach is that not all flagged data sets will necessarily be problematic because other factors, such as covariate adjustment in the original GWAS or deviations from HWE for reasons other than measurement error, could also cause deviations between expected and reported effect sizes. Therefore, SNPs flagged by this approach may still be suitable for downstream MR analyses.

A limitation of our comparative approach is that it may be less effective when there are zero, or few, known genetic associations for the trait of interest. This could happen, for example, when working with understudied or rare characteristics, for which existing published GWAS may be underpowered. In such a situation, comparisons with genetic associations for closely related traits could still be informative. Alternatively, there are a growing number of online platforms that collate summary data from multiple GWAS, which in principle could also be considered as reference data sets when the trait of interest is absent from the GWAS catalogue. These include OpenGWAS (https://gwas.mrcieu.ac.uk/), GWAS ATLAS (https://atlas.ctglab.nl/), GWAS Central (https://www.gwascentral.org/), PhenoScanner (http://www.phenoscanner.medschl.cam.ac.uk/) and Global Biobank Engine (https://biobankengine.stanford.edu/).

We manually mapped the text descriptions for each cancer type to the EFO, which could be inefficient when working with hundreds or thousands of traits. A more efficient approach would be to use the EMBL-EBI Zooma (https://www.ebi.ac.uk/spot/zooma) ontology mapping service, which supports command line access via a REST API.

### Two-sample population assumption

One of the key assumptions made in two-sample MR is that the studies used to define the exposure and the outcome come from the same population. The comparison of allele frequencies between test data sets and reference populations can in principle be used to evaluate this assumption. For example, in our own analyses, allele frequencies in the European origin cancer studies and 1000 genomes European super population were consistently strongly correlated (the same applied to the East Asian origin studies and the 1000 genomes East Asian super population), indicating that the reported study ancestries were broadly accurate. However, our QC procedure was not designed to specifically test for ancestral origins and was restricted to SNPs with a narrow allele frequency range. A more efficient approach would be to select SNPs with a much wider range of variation in minor allele frequency than chosen here. The need to assess the ‘same population’ assumption is becoming more urgent with the growing diversity of GWAS, including a growing number of trans-ethnic and admixed studies.

## Conclusion

We have developed a QC pipeline that can be used to flag metadata and summary-data errors and a range of analytical issues in GWAS results, which in turn can be used to enhance the integrity of downstream two-sample MR analyses. We applied the pipeline to the FAMRC, identifying errors with potential to introduce substantial bias in seven studies. After resolving analytical issues and excluding problematic studies, 160 data sets from 51 studies were retained, representing 90 unique cancer types generated in analyses of 566 665 cancer cases and 1 622 374 controls. The methods developed here are available to other researchers via the CheckSumStats R package (https://github.com/MRCIEU/CheckSumStats).

## Supplementary Material

Supplementary figures

Supplementary material

Supplementary tables

## Figures and Tables

**Table 1:** Cancer studies in the Fatty Acids in Cancer Mendelian Randomization Collaboration

Cancer	Contributing studies	Organ site/cell	Cases	Controls	Population
Overall/pan cancer					
Cancer (all-cause)	UKB; FinnGen	Multiple	101 440	437 298	European
Cancer (excluding non-melanoma skin cancer)	UKB	Multiple	50 643	372 016	European
Blood cancers					
Acute lymphoblastic leukaemia	BC-ALL; C-ALL; SJ-COG	B lymphocytes; lymphocytes	3178	33 048	European
B cell non-Hodgkin lymphoma	BC-NHL	B lymphocytes	253	1438	East Asian
Blood cancer	UKB; BJ; FinnGen	Leukocytes	6789	678 731	European and East Asian
Chronic lymphocytic leukaemia	InterLymph	B lymphocytes	3100	7667	European
Chronic myeloid leukaemia	KCML	Myeloid cells	201	497	East Asian
Diffuse large B cell lymphoma	InterLymph	B lymphocytes	3857	7666	European
Follicular lymphoma	InterLymph; FinnGen	B lymphocytes	3005	104 448	European
Hodgkin’s lymphoma	HLS	Lymphocytes	3077	13 680	European
Leukaemia	UKB	Leukocytes	1260	372 016	European
Lymphoid leukaemia	UKB; FinnGen	Lymphocytes	958	468 317	European
Lymphoma	UKB	Lymphocytes	1752	359 442	European
Marginal zone lymphoma	InterLymph	B lymphocytes	825	6221	European
Multiple myeloma	MMS; UKB; FinnGen	Plasma cells	2495	478 726	European
Myeloid leukaemia	UKB	Myeloid cells	462	372 016	European
Non-follicular lymphoma	FinnGen	Lymphocytes	344	96 155	European
Non-Hodgkin lymphoma unspecified	FinnGen	Lymphocytes	155	96 344	European
Digestive system cancers					
Biliary tract cancer	BJ	Biliary tract	339	195 745	East Asian
Cancer of digestive organs	UKB; FinnGen	Digestive organs	7272	450 421	European
Colon cancer	GECCO/CORECT/CCFR	Bowel	31 083	67 694	European
Colorectal cancer	GECCO/CORECT/CCFR; ACCC; FinnGen	Bowel	82 546	211 703	European and East Asian
Colorectal cancer in females	GECCO/CORECT/CCFR	Bowel	26 843	32 820	European
Colorectal cancer in males	GECCO/CORECT/CCFR	Bowel	31 288	34 527	European
Distal colorectal cancer	GECCO/CORECT/CCFR	Bowel	15 306	67 694	European
Oesophageal adenocarcinoma	EAS; UKB	Oesophagus	4852	389 175	European
Oesophageal squamous cell carcinoma	N-UGC; BJ	Oesophagus	3313	198 446	East Asian
Gastric adenocarcinoma	BJ; N-UGC	Stomach	8913	198 453	East Asian
Gastric cardia adenocarcinoma	N-UGC	Stomach	1189	2708	East Asian
Liver and bile duct cancer	UKB	Liver	350	372 016	European
Liver cancer	BJ; CHC; UKB; HKHC	Liver	3667	569 323	East Asian and European
Non-cardia gastric adenocarcinoma	N-UGC; NB-UGC	Stomach	2033	4981	East Asian
Pancreatic cancer	PanC4; PanScan IÞII; PanScan III; BJ; FinnGen	Pancreas	9711	304 511	European and East Asian
Proximal colorectal cancer	GECCO/CORECT/CCER	Bowel	13 857	67 694	European
Rectal cancer	GECCO/CORECT/CCER	Bowel	15 775	67 694	European
Small bowel cancer	UKB	Small bowel	156	337 003	European
Endocrine cancers					
Endocrine gland cancer	FinnGen	Endocrine glands	328	96 171	European
Thyroid cancer	EPITHYR; TCS; UKB; FinnGen	Thyroid	2923	506 047	European
Skin cancers					
Basal cell carcinoma	23NMSC; UKB; HNMSC	Basal cells	21 477	745 697	European
Malignant non-melanoma skin cancer	UKB	Basal/squamous	23 694	372 016	European
Malignant skin cancer	UKB; FinnGen	NA	17 426	440 267	European
Melanoma	MMAC; UKB	Melanocytes	14 780	387 260	European
Squamous cell carcinoma	23NMSC; HNMSC; UKB	Squamous cells	7808	628 831	European
Nervous system cancers					
Brain cancer	UKB; FinnGen	Brain	748	468 373	European
Central nervous system and eye cancer	FinnGen	Brain	207	96 292	European
Glioma	GICC/MDA; GliomaScan; UCSF_Mayo; UCSF_AGS Þ SFAGS	Brain/glial cells	8624	12 985	European
Meningioma	MENC	Brain	1606	9823	European
Neuroblastoma	NBS	Neuroblasts	2101	4202	European
Uveal melanoma	UMS	Eye/melanocytes	259	401	European
Reproductive cancers					
Advanced prostate cancer	PRACTICAL	Prostate	15 167	58 308	European
Breast cancer	BCAC; UKB; FinnGen	Breast	139 445	398 407	European
Cervical cancer	MCCS; SCCS; BJ	Uterus	4505	100 160	European and East Asian
Clear cell ovarian cancer	OCAC	Ovary	1366	40 941	European
Early-onset prostate cancer	PRACTICAL	Prostate	6988	44 256	European
Endometrial cancer	ECAC; BJ; FinnGen	Uterus	14 271	252 606	European and East Asian
Endometrioid ovarian cancer	OCAC	Ovary	2810	40 941	European
ER– breast cancer	BCAC	Breast	21 468	105 974	European
ER– breast cancer	BCAC	Breast	69 501	105 974	European
Female genital cancer	FinnGen	Female genital organs	672	53 590	European
High-grade serous ovarian cancer	OCAC	Ovary	13 037	40 941	European
High risk breast cancer	KHBC	Breast	1478	5979	East Asian
Invasive mucinous ovarian cancer	OCAC	Ovary	1417	40 941	European
Low-grade and low malignant potential serous ovarian cancer	OCAC	Ovary	2966	40 941	European
Low-grade serous ovarian cancer	OCAC	Ovary	1012	40 941	European
Low malignant potential mucinous ovarian cancer	OCAC	Ovary	1149	40 941	European
Low malignant potential ovarian cancer	OCAC	Ovary	3103	40 941	European
Low malignant potential serous ovarian cancer	OCAC	Ovary	1954	40 941	European
Mucinous ovarian cancer	OCAC	Ovary	2566	40 941	European
Ovarian cancer	OCAC; OCAC (EAS); UKB; BJ; FinnGen	Ovary	30 869	387 356	European and East Asian
Prostate cancer	PRACTICAL; UKB; BJ; FinnGen	Prostate	95 512	378 951	European and East Asian
Serous ovarian cancer	OCAC	Ovary	14 049	40 941	European
Respiratory cancers					
Lung adenocarcinoma	ILCCO	Lung	11 245	54 619	European
Lung cancer	ILCCO; BJ; UKB; FinnGen	Lung	36 660	732 695	European and East Asian
Lung cancer in ever smokers	ILCCO	Lung	23 848	16 605	European
Lung cancer in never smokers	ILCCO	Lung	2303	6995	European
Nasopharyngeal carcinoma	TNC; MNC	Nasopharynx	548	741	East Asian
Oral cancer	INHANCE	Mouth and throat	2990	6585	European
Oral cavity and pharyngeal cancer	INHANCE; UKB; FinnGen	Mouth and throat	7359	474 866	European
Oropharyngeal cancer	INHANCE	Mouth and throat	2641	6585	European
Pleural mesothelioma	MPM	Lung	407	389	European
Respiratory and intrathoracic cancer	UKB; FinnGen	Respiratory and intrathoracic organs	2559	455 134	European
Small cell lung carcinoma	ILCCO	Lung	2791	20 580	European
Squamous cell lung cancer	ILCCO	Lung/squamous cells	7704	54 763	European
Urinary/other cancers					
Bladder cancer	NBCS; UKB; FinnGen	Bladder	3719	460 518	European
Kidney cancer	KidRISK; UKB; FinnGen	Kidney	12 199	578 500	European
Kidney cancer in females	KidRISK	Kidney	1992	3095	European
Kidney cancer in males	KidRISK	Kidney	3227	4916	European
Urinary tract cancer	UKB; FinnGen	Urinary organs	2531	455 162	European
Ewing’s sarcoma	ESS	bone	401	684	European

Further details of the studies, such as PubMed identifiers and explanations of study abbreviations, can be found in Supplementary Tables S5 and S6.

## Data Availability

Full GWAS summary data for the included cancer studies can be downloaded from Open GWAS (https://gwas.mrcieu.ac.uk/) or the GWAS catalogue FTP site (https://www.ebi.ac.uk/gwas/home), or obtained by direct correspondence with the relevant study. Supplementary Table S5 contains further details on the underlying source for each cancer GWAS summary-data set, such as Open GWAS identifiers and columns indicating whether the data were downloaded from the GWAS catalogue or obtained by correspondence. Full GWAS summary data for cancers generated in UK Biobank, under application number 15825, can be downloaded from either Open GWAS or the University of Bristol data repository at https://doi.org/10.5523/bris.aed0u12w0ede20olb0m77p4b9. Summary genetic data corresponding to the fatty acid SNP set (i.e. a subset of the full summary data) for all cancer studies can be found at the following github repository: https://github.com/mightyphil2000/fatty-acids/tree/master/outcome_data/data/harmonised.

## Box 1 General overview of Mendelian randomization studies

The main aim of MR is to assess the potentially causal nature and direction of associations between exposure and outcome traits. Rather than studying the exposure–outcome relationship directly, i.e. using phenotypically measured levels of the exposure, MR uses genetic polymorphisms as instruments or proxies for the exposure. If the genetic instrument for the exposure is associated with the outcome of interest, this can be taken as evidence for a causal effect of the exposure on the outcome, so long as instrumental variable (IV) assumptions are met: (i) the instrument is associated with the exposure; (ii) the instrument is not associated with confounders of the exposure–outcome association; and (iii) the instrument is associated with the outcome exclusively through its effect on the exposure. Although violations of assumptions can be introduced by genomic confounding or horizontal pleiotropy, an increasingly sophisticated range of sensitivity analyses are available that can be used to model the impact of such bias on MR findings.

In the two-sample approach to MR, genetic summary data for the exposure and the outcome are obtained from separate studies. This greatly increases the scope of MR, as it means the method can be applied to any disease case–control collection regardless of whether the exposure has been directly measured or not. The success of GWAS has greatly increased the number of traits with available genetic association or summary data. In principle, any heritable trait with summary genetic association data can be used to define an exposure or an outcome in a two-sample MR study and thus the scope for what counts as an exposure or an outcome is very broad. Exposure and outcome traits can vary from relatively simple molecular traits, such as expression or protein traits, to highly complex traits, such as human behaviours and disease outcomes.

## Key Messages

- Metadata errors (e.g. incorrect specification of the effect allele column) and other analytical issues can introduce substantial bias into Mendelian randomization (MR) studies but have received relatively little attention in comparison to other sources of bias, such as violations of instrument variable assumptions.
- We found that 11.6% of the studies in the Fatty Acids in Cancer Mendelian Randomization Collaboration were subject to metadata errors or analytical issues with the potential to introduce substantial bias into MR analyses (e.g. inferences of causal effect in the wrong direction or bias to the null).
- Previously developed guidelines for conducting MR studies provided insufficient safeguards against such errors.
- We developed additional guidelines and the CheckSumStats R package (https://github.com/MRCIEU/CheckSumStats) that can reliably identify and correct metadata errors and other analytical issues at the study design stage.

## References

[1] Zheng J, Baird D, Borges M-C (2017). Recent developments in Mendelian randomization studies. Curr Epidemiol Rep.

[2] Burgess S, Small DS, Thompson SG (2017). A review of instrumental variable estimators for Mendelian randomization. Stat Methods Med Res.

[3] Haycock PC, Burgess S, Wade KH, Bowden J, Relton C, Davey Smith G (2016). Best (but oft-forgotten) practices: the design, analysis, and interpretation of Mendelian randomization studies. Am J Clin Nutr.

[4] Hartwig FP, Davies NM, Hemani G, Smith GD (2016). Counterfactual causation: Avoiding the downsides of a powerful, widely applicable but potentially fallible technique. Int J Epidemiol.

[5] Lyon MS, Andrews SJ, Elsworth B, Gaunt TR, Hemani G, Marcora E (2021). The variant call format provides efficient and robust storage of GWAS summary statistics. Genome Biol.

[6] Zheng J, Haberland V, Baird D (2020). Phenome-wide Mendelian randomization mapping the influence of the plasma proteome on complex diseases. Nat Genet.

[7] Kazmi N, Haycock P, Tsilidis K, PRACTICAL Consortium, CRUK, BPC3, CAPS, PEGASUS (2020). Appraising causal relationships of dietary, nutritional and physical-activity exposures with overall and aggressive prostate cancer: two-sample Mendelian-randomization study based on 79 148 prostate-cancer cases and 61 106 controls. Int J Epidemiol.

[8] Saunders CN, Cornish AJ, Kinnersley B, Collaborators (2021). Searching for causal relationships of glioma: a phenome-wide Mendelian randomisation study. Br J Cancer.

[9] Yuan S, Larsson SC (2020). An atlas on risk factors for type 2 diabetes: a wide-angled Mendelian randomisation study. Diabetologia.

[10] Haycock PC, Burgess S, Nounu A, Telomeres Mendelian Randomization Collaboration (2017). Association between telomere length and risk of cancer and non-neoplastic diseases. JAMA Oncol.

[11] Anderson CA, Pettersson FH, Clarke GM, Cardon LR, Morris AP, Zondervan KT (2010). Data quality control in genetic case-control association studies. Nat Protoc.

[12] Winkler TW, Day FR, Croteau-Chonka DC, Genetic Investigation of Anthropometric Traits (GIANT) Consortium (2014). Quality control and conduct of genome-wide association meta-analyses. Nat Protoc.

[13] Buniello A, Macarthur JAL, Cerezo M (2019). The NHGRI-EBI GWAS Catalog of published genome-wide association studies, targeted arrays and summary statistics 2019. Nucleic Acids Res.

[14] Elsworth B, Lyon M, Alexander T (2020). The MRC IEU OpenGWAS data infrastructure. bioRxiv.

[15] Hemani G ieugwasr: R interface to the IEU GWAS database API.

[16] Ishigaki K, Akiyama M, Kanai M (2020). Large-scale genome-wide association study in a Japanese population identifies novel susceptibility loci across different diseases. Nat Genet.

[17] Tanikawa C, Kamatani Y, Takahashi A (2018). GWAS identifies two novel colorectal cancer loci at 16q24.1 and 20q13.12. Carcinogenesis.

[18] Nagai A, Hirata M, Kamatani Y, BioBank Japan Cooperative Hospital Group (2017). Overview of the BioBank Japan project: study design and profile. J Epidemiol.

[19] Ruth M, Gibran H, Tom D, Laura C, Harrison S, Paternoster L UK Biobank Genetic Data: MRC-IEU Quality Control, version 2—Datasets—databris.

[20] Ruth M, Elsworth BL, Raistrick CA, Paternoster L, Hemani G, Gaunt T MRC IEU UK Biobank GWAS pipeline version 2—Datasets—databris.

[21] Malone J, Holloway E, Adamusiak T (2010). Modeling sample variables with an Experimental Factor Ontology. Bioinformatics.

[22] Wickham H (2016). Ggplot2: Elegant Graphics for Data Analysis.

[23] Durinck S, Moreau Y, Kasprzyk A (2005). BioMart and Bioconductor: a powerful link between biological databases and microarray data analysis. Bioinformatics.

[24] Durinck S, Spellman PT, Birney E, Huber W (2009). Mapping identi-fiers for the integration of genomic datasets with the R/ Bioconductor package biomaRt. Nat Protoc.

[25] Auguie B gridExtra: Miscellaneous Functions for ‘Grid’ Graphics R package version 23.

[26] Wilke CO cowplot: Streamlined Plot Theme and Plot Annotations for ‘ggplot2’.

[27] Auguie B gridExtra: Miscellaneous Functions for ‘Grid’ Graphics.

[28] Henry L, Wickham H purrr: Functional Programming Tools.

[29] Wickham H, François R, Henry L, Müller K dplyr: A Grammar of Data Manipulation.

[30] Müller K, Wickham H tibble: Simple Data Frames.

[31] Bache SM, Wickham H magrittr: A Forward-Pipe Operator for R.

[32] Magno R, Maia A-TT, Wren J (2020). gwasrapidd: an R package to query, download and wrangle GWAS Catalog data. Bioinformatics.

[33] Guan W, Steffen BT, Lemaitre RN (2014). Genome-wide association study of plasma n6 polyunsaturated fatty acids within the cohorts for heart and aging research in genomic epidemiology consortium. Circ Cardiovasc Genet.

[34] Dorajoo R, Sun Y, Han Y (2015). A genome-wide association study of n-3 and n-6 plasma fatty acids in a Singaporean Chinese population. Genes Nutr.

[35] Zhu J, Manichaikul A, Hu Y (2017). Meta-analysis of genome-wide association studies identifies three novel loci for saturated fatty acids in East Asians. Eur J Nutr.

[36] Tintle NL, Pottala JV, Lacey S (2015). A genome-wide association study of saturated, mono- and polyunsaturated red blood cell fatty acids in the Framingham Heart Offspring Study. Prostaglandins Leukot Essent Fatty Acids.

[37] Kettunen J, Demirkan A, Würtz P (2016). Genome-wide study for circulating metabolites identifies 62 loci and reveals novel systemic effects of LPA. Nat Commun.

[38] Shin S-Y, Fauman EB, Petersen A-K, Multiple Tissue Human Expression Resource (MuTHER) Consortium (2014). An atlas of genetic influences on human blood metabolites. Nat Genet.

[39] Auton A, Brooks LD, Durbin RM, 1000 Genomes Project Consortium (2015). A global reference for human genetic variation. Nature.

[40] Harrison S Estimating an Odds Ratio from a GWAS only reporting the P value—Sean Harrison: Blog.

[41] Loh PR, Kichaev G, Gazal S, Schoech AP, Price AL (2018). Mixed-model association for biobank-scale datasets. Nat Genet.

[42] Lemaitre RN, King IB, Kabagambe EK (2015). Genetic loci associated with circulating levels of very long-chain saturated fatty acids. J Lipid Res.

[43] de Oliveira Otto MC, Lemaitre RN, Sun Q, Loor JJ (2018). Genome-wide association meta-analysis of circulating odd-numbered chain saturated fatty acids: results from the CHARGE Consortium. PLoS One.

[44] Tanaka T, Shen J, Abecasis GR, Georges M (2009). Genome-wide association study of plasma polyunsaturated fatty acids in the InCHIANTI Study. PLoS Genet.

[45] Gieger C, Geistlinger L, Altmaier E, Gibson G (2008). Genetics meets metabolomics: a genome-wide association study of metabolite profiles in human serum. PLoS Genet.

[46] Mychaleckyj JC, Nayak U, Colgate ER (2018). Multiplex genomewide association analysis of breast milk fatty acid composition extends the phenotypic association and potential selection of FADS1 variants to arachidonic acid, a critical infant micronutrient. J Med Genet.

[47] Hu Y, Tanaka T, Zhu J (2017). Discovery and fine-mapping of loci associated with MUFAs through trans-ethnic meta-analysis in Chinese and European populations. J Lipid Res.

[48] Mozaffarian D, Kabagambe EK, Johnson CO (2015). Genetic loci associated with circulating phospholipid trans fatty acids: a meta-analysis of genome-wide association studies from the CHARGE Consortium. Am J Clin Nutr.

[49] Lemaitre RN, Tanaka T, Tang W, McCarthy MI (2011). Genetic loci associated with plasma phospholipid n-3 fatty acids: a meta-analysis of genome-wide association studies from the CHARGE Consortium. PLoS Genet.

[50] Wu JHY, Lemaitre RN, Manichaikul A (2013). Genome-wide association study identifies novel loci associated with concentrations of four plasma phospholipid fatty acids in the de novo lipogenesis pathway: results from the Cohorts for Heart and Aging Research in Genomic Epidemiology (CHARGE) consortium. Circ Cardiovasc Genet.

[51] Veenstra J, Kalsbeek A, Westra J, Disselkoen C, Smith EC, Tintle N (2017). Genome-wide interaction study of omega-3 PUFAs and other fatty acids on inflammatory biomarkers of cardiovascular health in the Framingham Heart Study. Nutrients.

[52] Kettunen J, Tukiainen T, Sarin A-P (2012). Genome-wide association study identifies multiple loci influencing human serum metabolite levels. Nat Genet.

[53] Willer CJ, Li Y, Abecasis GR (2010). METAL: fast and efficient meta-analysis of genome wide association scans. Bioinformatics.

[54] Chahal HS, Wu W, Ransohoff KJ (2016). Genome-wide association study identifies 14 novel risk alleles associated with basal cell carcinoma. Nat Commun.

[55] Huyghe JR, Bien SA, Harrison TA (2019). Discovery of common and rare genetic risk variants for colorectal cancer. Nat Genet.

[56] Rajaraman P, Melin BS, Wang Z (2012). Genome-wide association study of glioma and meta-analysis. Hum Genet.

[57] Sud A, Thomsen H, Law PJ, PRACTICAL Consortium (2017). Genome-wide association study of classical Hodgkin lymphoma identifies key regulators of disease susceptibility. Nat Commun.

[58] Zhang M, Song F, Liang L (2013). Genome-wide association studies identify several new loci associated with pigmentation traits and skin cancer risk in European Americans. Hum Mol Genet.

[59] McKay JD, Hung RJ, Han Y, SpiroMeta Consortium (2017). Large-scale association analysis identifies new lung cancer susceptibility loci and heterogeneity in genetic susceptibility across histological subtypes. Nat Genet.

[60] Lesseur C, Diergaarde B, Olshan AF (2016). Genome-wide association analyses identify new susceptibility loci for oral cavity and pharyngeal cancer. Nat Genet.

[61] Berndt SI, Camp NJ, Skibola CF (2016). Meta-analysis of genome-wide association studies discovers multiple loci for chronic lymphocytic leukemia. Nat Commun.

[62] Cerhan JR, Berndt SI, Vijai J (2014). Genome-wide association study identifies multiple susceptibility loci for diffuse large B cell lymphoma. Nat Genet.

[63] Skibola CF, Berndt SI, Vijai J (2014). Genome-wide association study identifies five susceptibility loci for follicular lymphoma outside the HLA region. Am J Hum Genet.

[64] Vijai J, Wang Z, Berndt SI (2015). A genome-wide association study of marginal zone lymphoma shows association to the HLA region. Nat Commun.

[65] Chahal HS, Lin Y, Ransohoff KJ (2016). Genome-wide association study identifies novel susceptibility loci for cutaneous squa- mous cell carcinoma. Nat Commun.

[66] Lee J-Y, Kim J, Kim S-W (2018). BRCA1/2-negative, high-risk breast cancers (BRCAX) for Asian women: genetic susceptibility loci and their potential impacts. Sci Rep.

[67] Scelo G, Purdue MP, Brown KM (2017). Genome-wide association study identifies multiple risk loci for renal cell carcinoma. Nat Commun.

[68] Leo PJ, Madeleine MM, Wang S (2017). Defining the genetic susceptibility to cervical neoplasia: a genome-wide association study. PLoS Genet.

[69] Claus EB, Cornish AJ, Broderick P (2018). Genome-wide association analysis identifies a meningioma risk locus at 11p15.5. Neuro Oncol.

[70] Swaminathan B, Thorleifsson G, Jöud M (2015). Variants in ELL2 influencing immunoglobulin levels associate with multiple myeloma. Nat Commun.

[71] Chin Y-M, Tan LP, Abdul Aziz N, Malaysian NPC Study Group (2016). Integrated pathway analysis of nasopharyngeal carcinoma implicates the axonemal dynein complex in the Malaysian cohort. Int J Cancer.

[72] Matullo G, Guarrera S, Betti M (2013). Genetic variants associated with increased risk of malignant pleural mesothelioma: a genome-wide association study. PLoS One.

[73] Wu C, Wang Z, Song X (2014). Joint analysis of three genome-wide association studies of esophageal squamous cell carcinoma in Chinese populations. Nat Genet.

[74] Hu N, Wang Z, Song X (2016). Genome-wide association study of gastric adenocarcinoma in Asia: a comparison of associations between cardia and non-cardia tumours. Gut.

[75] Wang Z, Dai J, Hu N (2017). Identification of new susceptibility loci for gastric non-cardia adenocarcinoma: pooled results from two Chinese genome-wide association studies. Gut.

[76] Lu Y, Kweon S-S, Cai Q (2020). Identification of novel loci and new risk variant in known loci for colorectal cancer risk in East Asians. Cancer Epidemiol Biomarkers Prev a Prev.

[77] Rafnar T, Vermeulen SH, Sulem P (2011). European genome-wide association study identifies SLC14A1 as a new urinary bladder cancer susceptibility gene. Hum Mol Genet.

[78] McDaniel LD, Conkrite KL, Chang X (2017). Common variants upstream of MLF1 at 3q25 and within CPZ at 4p16 associated with neuroblastoma. PLoS Genet.

[79] Phelan CM, Kuchenbaecker KB, Tyrer JP, OPAL study group (2017). Identification of 12 new susceptibility loci for different histotypes of epithelial ovarian cancer. Nat Genet.

[80] Lawrenson K, Song F, Hazelett DJ, Australian Ovarian Cancer Study Group (2019). Genome-wide association studies identify susceptibility loci for epithelial ovarian cancer in east Asian women. Gynecol Oncol.

[81] Klein AP, Wolpin BM, Risch HA (2018). Genome-wide meta-analysis identifies five new susceptibility loci for pancreatic cancer. Nat Commun.

[82] Schumacher FR, Al Olama AA, Berndt SI, Genetic Associations and Mechanisms in Oncology (GAME-ON)/Elucidating Loci Involved in Prostate Cancer Susceptibility (ELLIPSE) Consortium (2018). Association analyses of more than 140,000 men identify 63 new prostate cancer susceptibility loci. Nat Genet.

[83] Chen D, Juko-Pecirep I, Hammer J (2013). Genome-wide association study of susceptibility loci for cervical cancer. J Natl Cancer Inst.

[84] Trevin~o LR, Yang W, French D (2009). Germline genomic variants associated with childhood acute lymphoblastic leukemia. Nat Genet.

[85] Tse K-PP, Su W-HH, Chang K-PP (2009). Genome-wide association study reveals multiple nasopharyngeal carcinoma-associated loci within the HLA region at chromosome 6p21.3. Am J Hum Genet.

[86] Melin BS, Barnholtz-Sloan JS, Wrensch MR, GliomaScan Consortium (2017). Genome-wide association study of glioma subtypes identifies specific differences in genetic susceptibility to glioblastoma and non-glioblastoma tumors. Nat Genet.

[87] Vijayakrishnan J, Studd J, Broderick P, The PRACTICAL Consortium (2018). Genome-wide association study identifies susceptibility loci for B-cell childhood acute lymphoblastic leukemia. Nat Commun.

[88] Zhou W, Nielsen JB, Fritsche LG (2018). Efficiently controlling for case-control imbalance and sample relatedness in large-scale genetic association studies. Nat Genet.

[89] Mobuchon L, Battistella A, Bardel C (2017). A GWAS in uveal melanoma identifies risk polymorphisms in the CLPTM1L locus. NPJ Genomic Med.

[90] Mitchell JA, Warner TD (2006). COX isoforms in the cardiovascular system: understanding the activities of non-steroidal anti-inflammatory drugs. Nat Rev Drug Discov.

[91] Köhler A, Chen B, Gemignani F (2013). Genome-wide association study on differentiated thyroid cancer. J Clin Endocrinol Metab.

[92] Tan DEK, Foo JN, Bei J-X (2013). Genome-wide association study of B cell non-Hodgkin lymphoma identifies 3q27 as a susceptibility locus in the Chinese population. Nat Genet.

[93] Kim DHD, Lee S-T, Won H-H (2011). A genome-wide association study identifies novel loci associated with susceptibility to chronic myeloid leukemia. Blood.

[94] Law MH, Bishop DT, Lee JE, ATHENS Melanoma Study Group (2015). Genome-wide meta-analysis identifies five new susceptibility loci for cutaneous malignant melanoma. Nat Genet.

[95] Truong T, Lesueur F, Sugier PE (2021). Multiethnic genome-wide association study of differentiated thyroid cancer in the EPITHYR consortium. Int J Cancer.

[96] Li WQ, Hu N, Hyland PL (2013). Genetic variants in DNA repair pathway genes and risk of esophageal squamous cell carcinoma and gastric adenocarcinoma in a Chinese population. Carcinogenesis.

[97] Ciampa J, Yeager M, Amundadottir L (2011). Large-scale exploration of gene-gene interactions in prostate cancer using a multistage genome-wide association study. Cancer Res.

[98] Michailidou K, Lindström S, Dennis J, NBCS Collaborators (2017). Association analysis identifies 65 new breast cancer risk loci. Nature.

[99] Ellinghaus E, Stanulla M, Richter G (2012). Identification of germline susceptibility loci in ETV6-RUNX1-rearranged childhood acute lymphoblastic leukemia. Leukemia.

[100] Li S, Qian J, Yang Y (2012). GWAS identifies novel susceptibility loci on 6p21.32 and 21q21.3 for hepatocellular carcinoma in chronic hepatitis B virus carriers. PLoS Genet.

[101] Gharahkhani P, Fitzgerald RC, Vaughan TL, Wellcome Trust Case Control Consortium 2 (WTCCC2) (2016). Genome-wide association studies in oesophageal adenocarcinoma and Barrett’s oesophagus: a large-scale meta-analysis. Lancet Oncol.

[102] O’Mara TA, Glubb DM, Amant F (2018). Identification of nine new susceptibility loci for endometrial cancer. Nat Commun.

[103] Inoshita M, Numata S, Tajima A (2016). A significant causal association between C-reactive protein levels and schizophrenia. Sci Rep.

[104] Prins BP, Abbasi A, Wong A, PAGE Consortium (2016). Investigating the causal relationship of C-reactive protein with 32 complex somatic and psychiatric outcomes: a large-scale cross-consortium Mendelian randomization study. PLoS Med.

[105] Masuda T, Low SK, Akiyama M (2020). GWAS of five gynecologic diseases and cross-trait analysis in Japanese. Eur J Hum Genet.

[106] Rueger S, McDaid A, Kutalik Z (2018). Evaluation and application of summary statistic imputation to discover new height-associated loci. PLoS Genet.

[107] Skrivankova VW, Richmond RC, Woolf BAR (2021). Strengthening the reporting of observational studies in epidemiology using mendelian randomisation (STROBE-MR): explanation and elaboration. BMJ.

[108] Burgess S, Davey Smith G, Davies NM (2020). Guidelines for performing Mendelian randomization investigations. Wellcome Open Res.

