# Supplementary material for: Design and quality control of large-scale two-sample Mendelian randomization studies
Source: Int J Epidemiol. Author manuscript; Available in PMC 2024 Jan 8. (PMC10555669; doi:10.1093/ije/dyad018)
Supplement: Supplementary figures [file EMS190936-supplement-Supplementary_figures.docx]

Supplementary figure S1. Fatty acid search strategy and quality control procedure

**CHARGE**, Cohorts for Heart and Aging Research in Genomic Epidemiology Consortium; **FHS**, the Framingham study; **GWAS**, genome-wide association study; **NHAPC/MESA-CHI**, Nutrition and Health of Aging Population in China / Multi-Ethnic Study of Atherosclerosis - Chinese ancestry participants; **SCHS**, the Singapore Chinese Health Study; **TwinsUK/KORA**, Twins United Kingdom / Cooperative Health Research in the Region of Augsburg

Supplementary figure S2. Quality control report for genetic summary data from a genome-wide association of arachidonic acid in the Framingham heart study


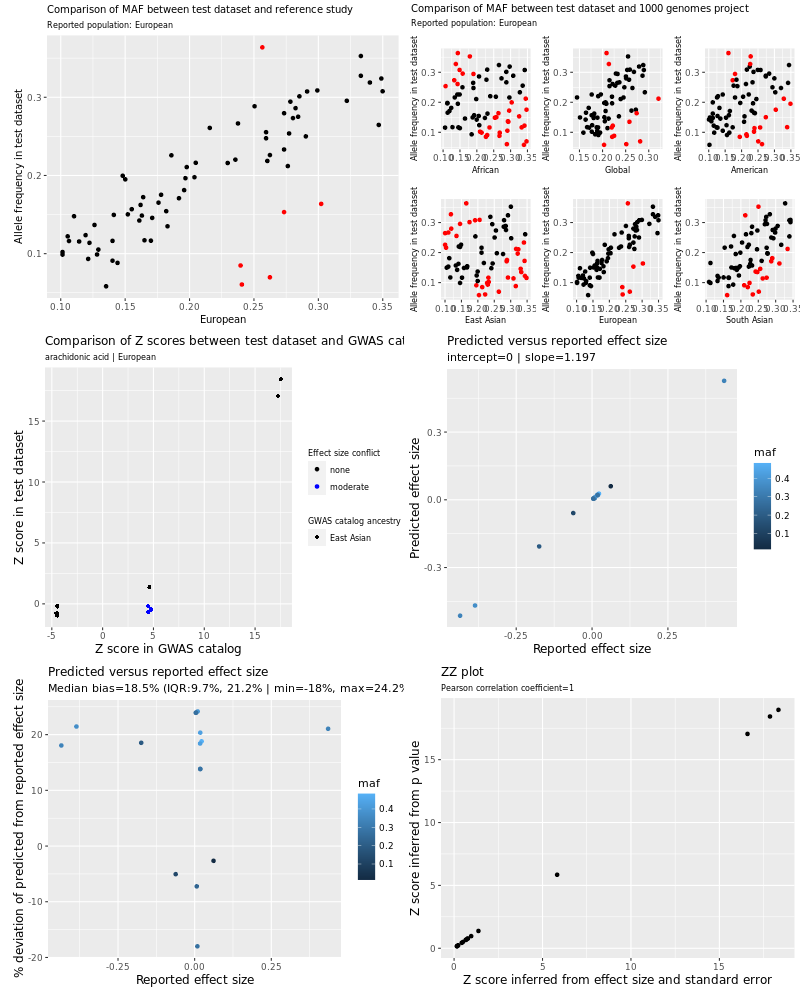


In the allele frequency plots, each red data point corresponds to SNPs with high allele frequency conflicts, due to deviation from the reference allele frequency by more than 10 points; GWAS, genome-wide association study; IQR, interquartile range; MAF, minor allele frequency

Supplementary figure S3. Quality control report for genetic summary data from a genome-wide association of linoleic acid the Kettunen study


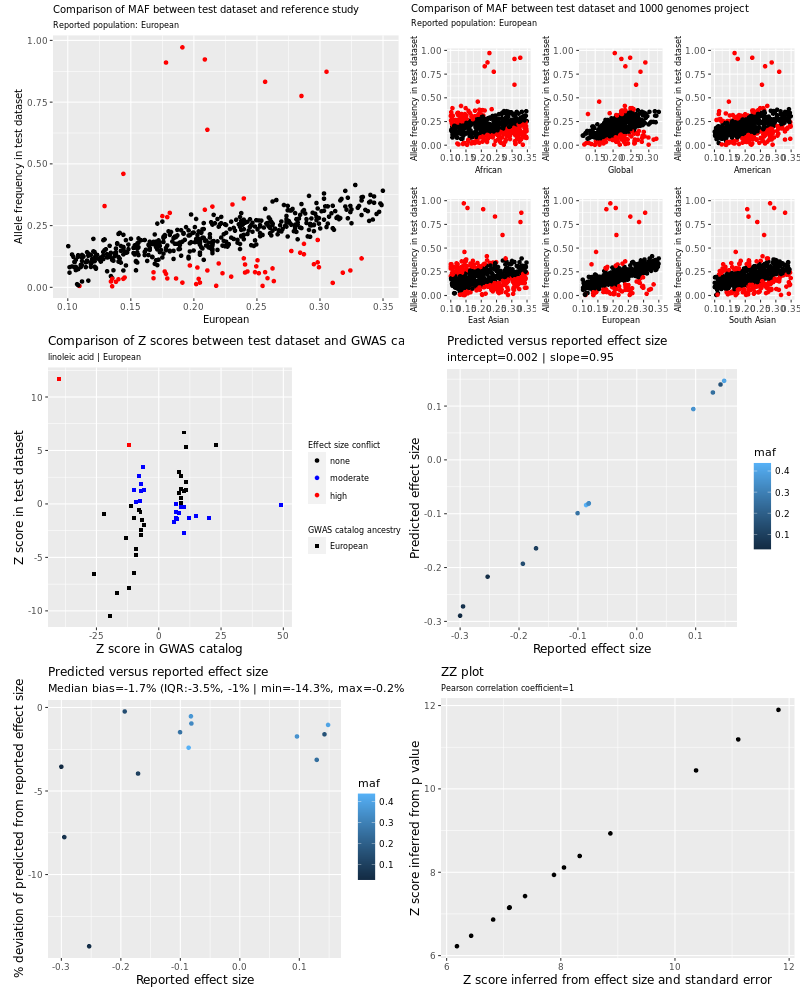


In the allele frequency plots, each red data point corresponds to SNPs with high allele frequency conflicts, due to having an allele frequency that is greater than 0.58 (when it is expected to be less than 0.5) or to deviation from the reference allele frequency by more than 10 points. GWAS, genome-wide association study; IQR, interquartile range; MAF, minor allele frequency

Supplementary figure S4. Quality control report for genetic summary data from a genome-wide association of arachidonic acid in the TwinsUK/KORA study


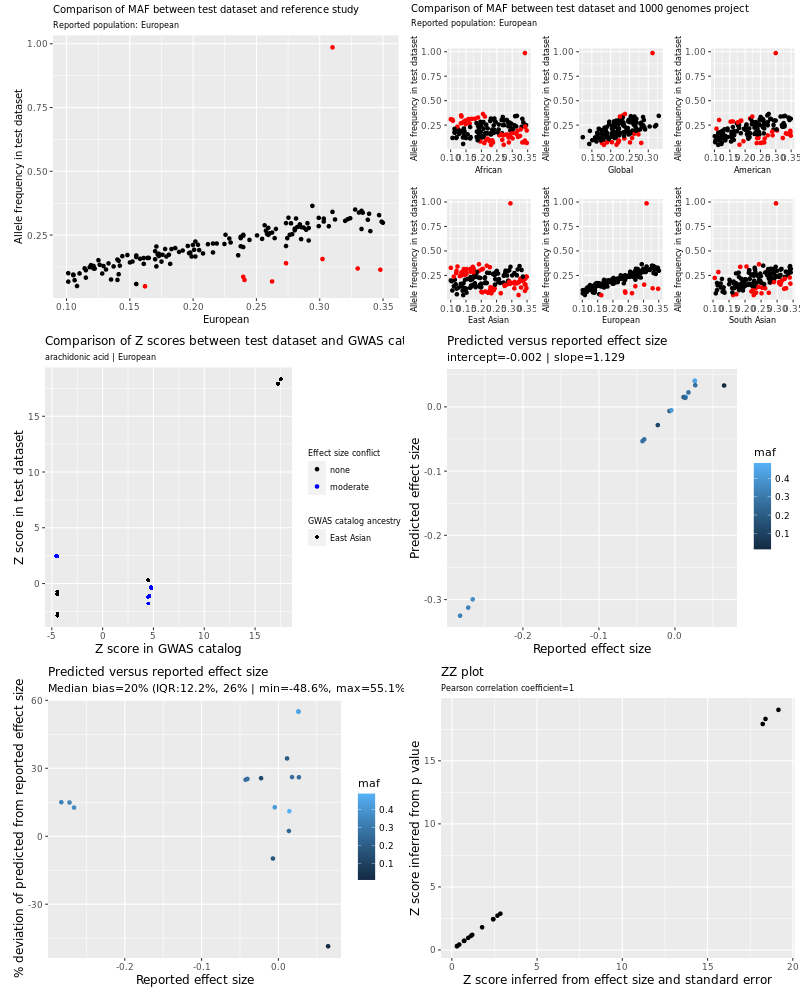


In the allele frequency plots, each red data point corresponds to SNPs with high allele frequency conflicts, due to having an allele frequency that is greater than 0.58 (when it is expected to be less than 0.5) or to deviation from the reference allele frequency by more than 10 points. GWAS, genome-wide association study; IQR, interquartile range; MAF, minor allele frequency

Supplementary figure S5. Quality control report for genetic summary data from a genome-wide association of arachidonic acid in the SCHS


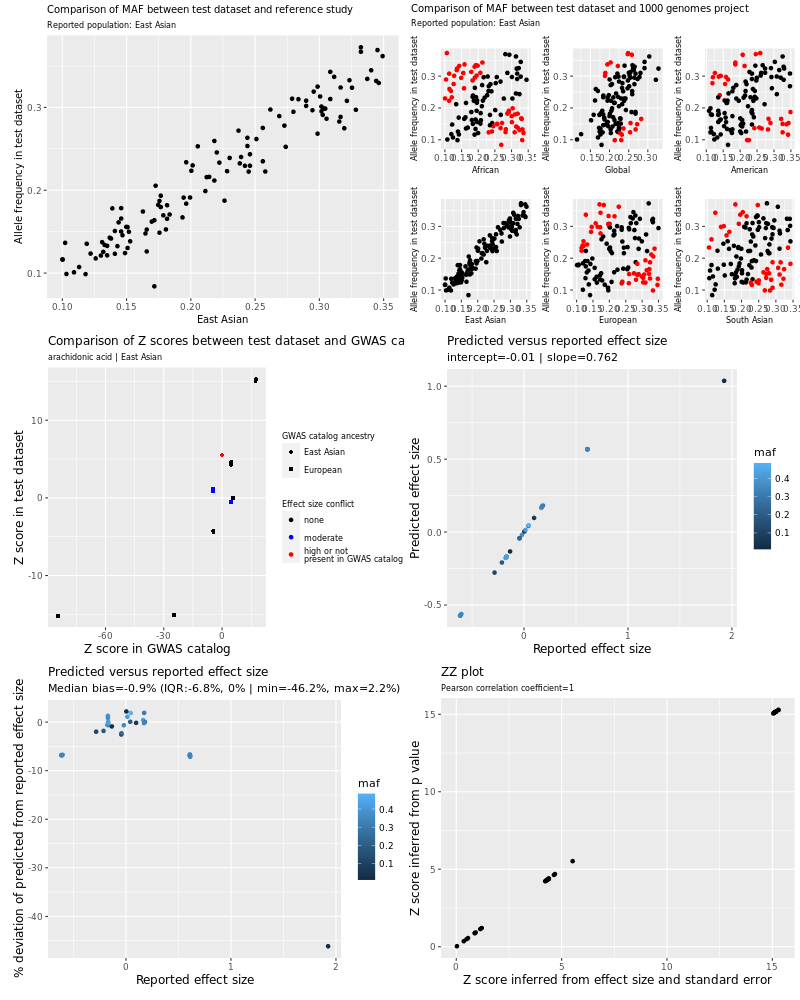


In the allele frequency plots, each red data point corresponds to SNPs with high allele frequency conflicts, due to deviation from the reference allele frequency by more than 10 points. GWAS, genome-wide association study; IQR, interquartile range; MAF, minor allele frequency

Supplementary figure S6. Quality control report for genetic summary data from a genome-wide association of stearic acid in the NHAPC/MESA-CHI study


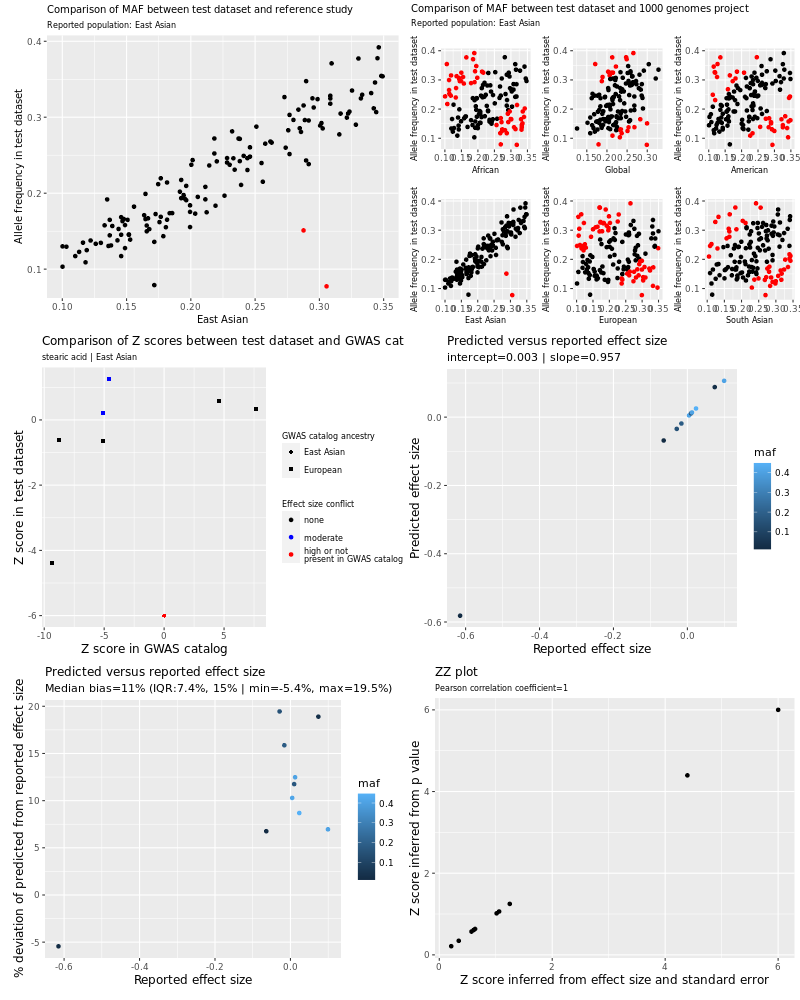


In the allele frequency plots, each red data point corresponds to SNPs with high allele frequency conflicts, due to deviation from the reference allele frequency by more than 10 points. GWAS, genome-wide association study; IQR, interquartile range; MAF, minor allele frequency

Supplementary figure S7. Relationship between reported and expected effect sizes for SNPs associated with arachidonic acid in CHARGE, before and after filtering out low quality SNPs

After exclusion of low quality SNPs

Before exclusion of low quality SNPs


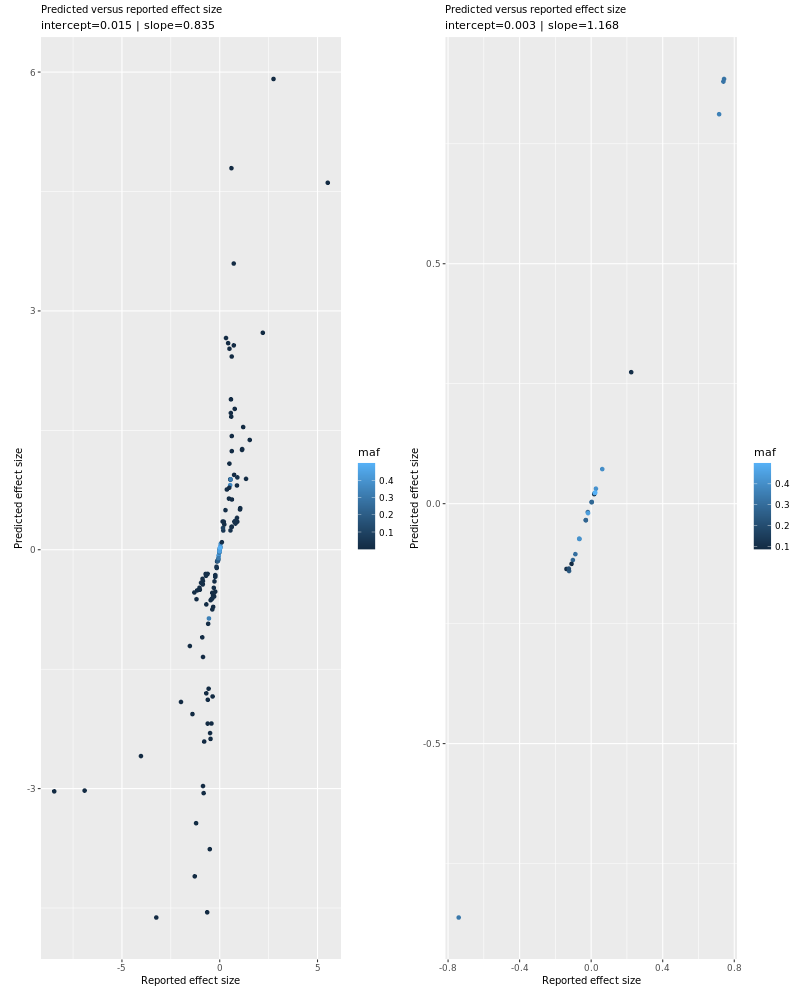


CHARGE, Cohorts for Heart and Aging Research in Genomic Epidemiology Consortium; maf, minor allele frequency; SNP, single nucleotide polymorphism

Supplementary figure S8. Application of a quality control pipeline to 166 datasets from 54 genome-wide association studies of cancer

Supplementary figure S9. Quality control report for genetic summary data from a genome-wide association of B cell non-Hodgkin lymphoma in the BC-NHL study (dataset ID 5)


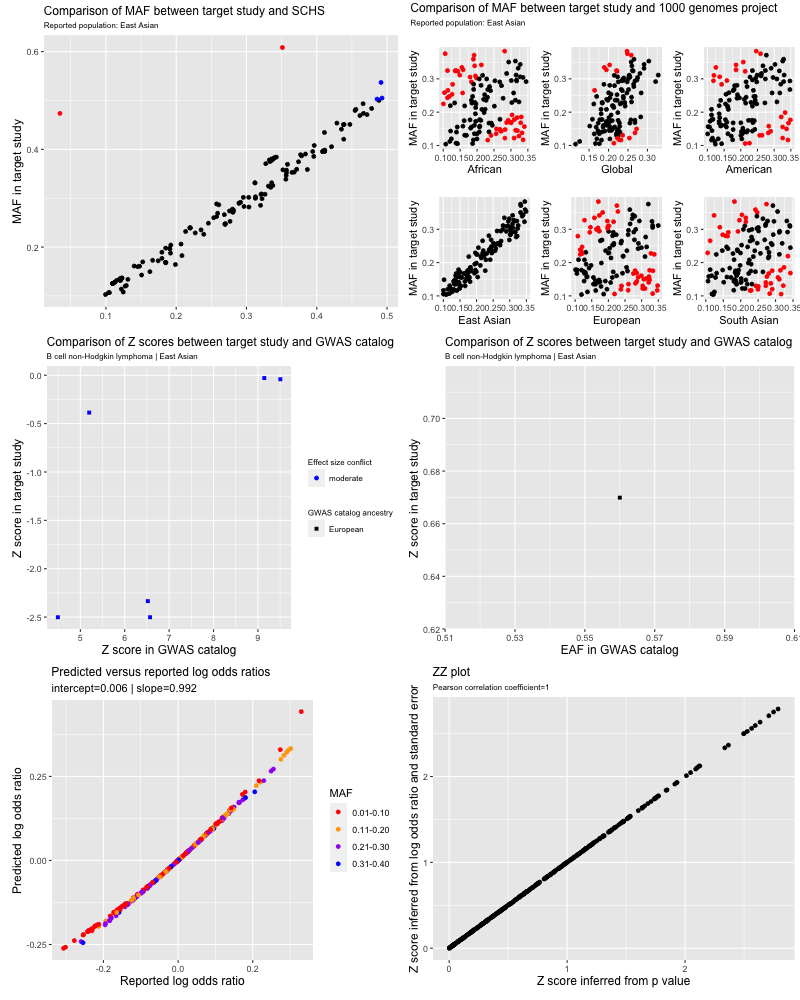


In the allele frequency subplots, each red data point corresponds to SNPs with high allele frequency conflicts, due to having an allele frequency that is greater than 0.58 (when it is expected to be less than 0.5) or to deviation from the reference allele frequency by more than 10 points. BC-NHL, B Cell Non-Hodgkin Lymphoma Study; EAF, effect allele frequency; GWAS, genome-wide association study; MAF, minor allele frequency; SCHS, Singapore Chinese Health Study

Supplementary figure S10. Quality control report for genetic summary data from a genome-wide association of neuroblastoma in the NBS dataset (ID 106)


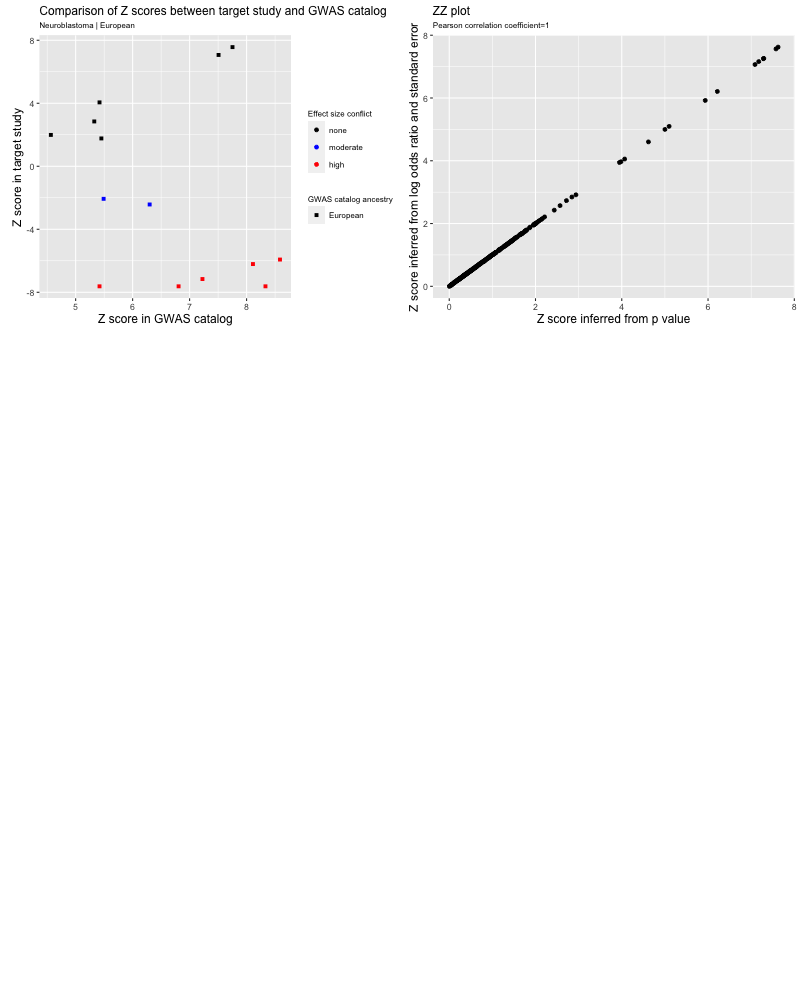


GWAS, genome-wide association study; NBS, Neuroblastoma Study

Supplementary figure S11. Quality control report for genetic summary data from a genome-wide association of glioma in the UCSF_AGS/SFAGS dataset (ID 133)


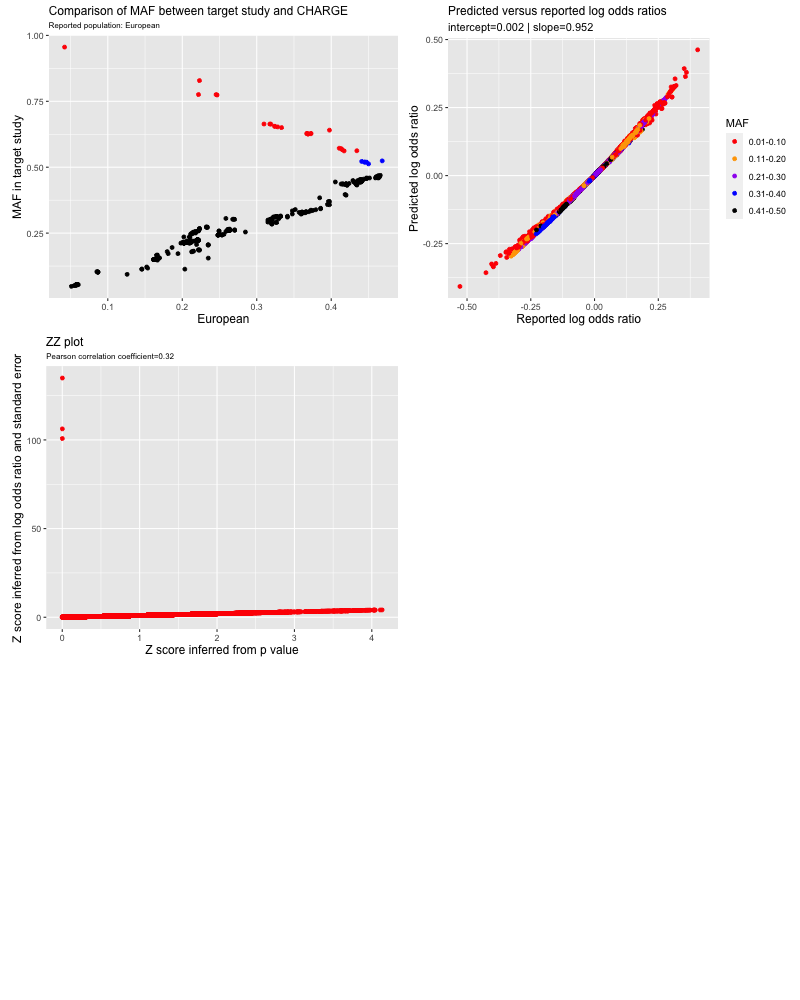


In the allele frequency subplots, each red data point corresponds to SNPs with high allele frequency conflicts, due to having an allele frequency that is greater than 0.58 (when it is expected to be less than 0.5) or to deviation from the reference allele frequency by more than 10 points; CHARGE, Cohorts for Heart and Aging Research in Genomic Epidemiology Consortium; MAF, minor allele frequency; UCSF_AGS/SFAGS, UCSF Adult Glioma Study / San Francisco Adult Glioma Study

Supplementary figure S12. Quality control report for genetic summary data from a genome-wide association study of nasopharyngeal carcinoma in the TNC dataset (ID=132)


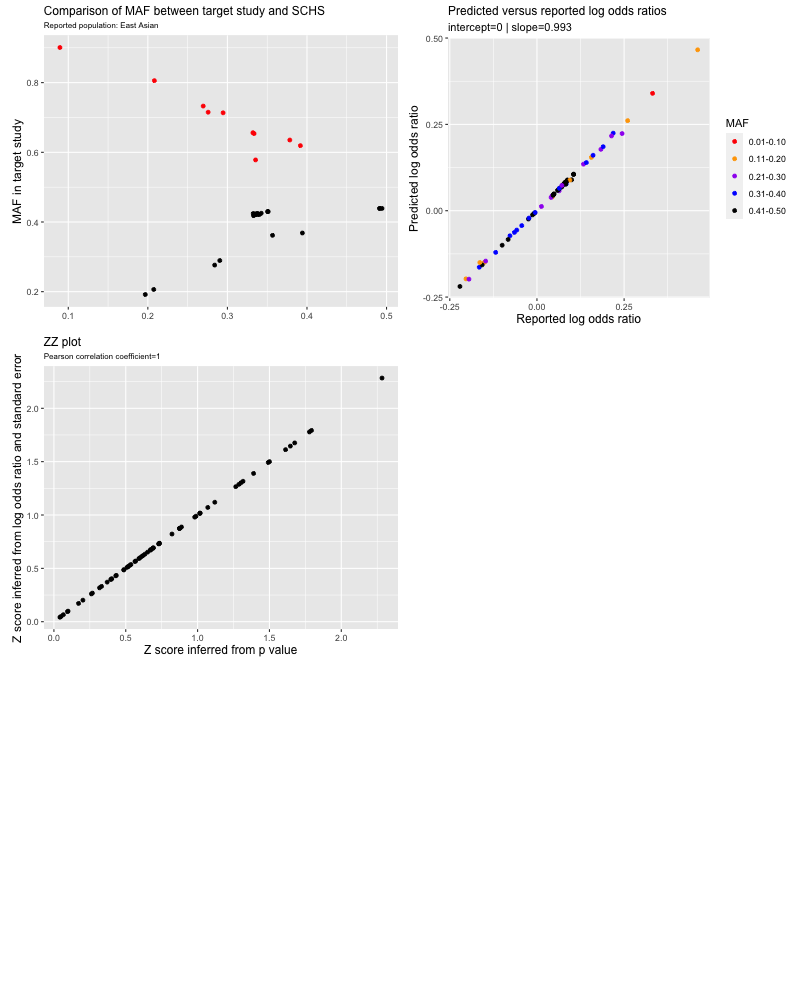


In the allele frequency subplots, each red data point corresponds to SNPs with high allele frequency conflicts, due to having an allele frequency that is greater than 0.58 (when it is expected to be less than 0.5) or to deviation from the reference allele frequency by more than 10 points; MAF, minor allele frequency; SCHS, the Singapore Chinese Health Study; TNC, Taiwan Nasopharyngeal Carcinoma Study

Supplementary figure S13a. Comparison of effect sizes between cancer datasets (ID1-ID83) and the NHGRI-EBI GWAS catalog


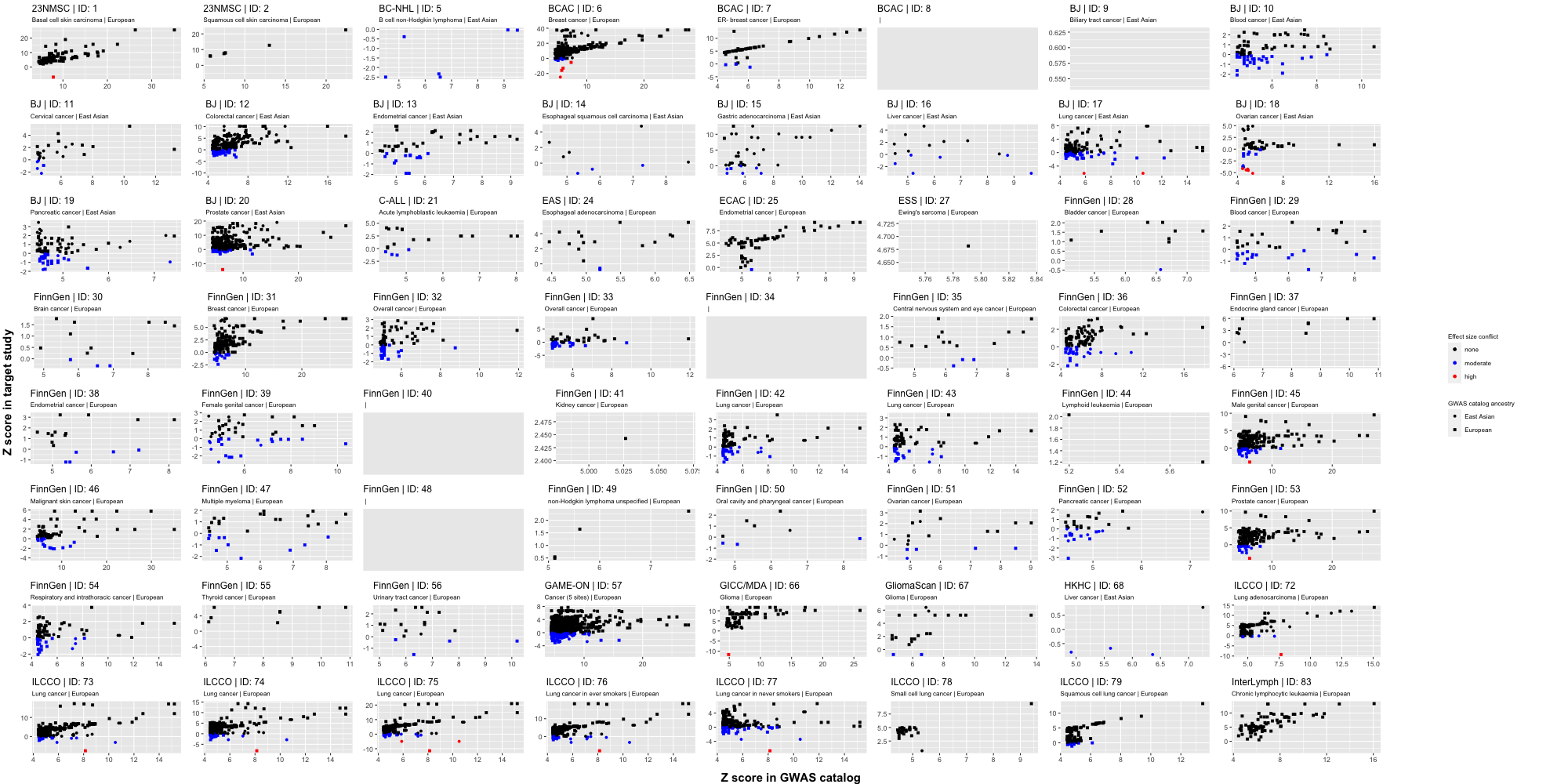


Study abbreviations are explained in supplementary table 6. EBI, European Bioinformatics Institute; GWAS, genome-wide association study; NHGRI, National Human Genome Research Institute

Supplementary figure S13b. Comparison of effect sizes between cancer datasets (ID84-ID1499) and the NHGRI-EBI GWAS catalog


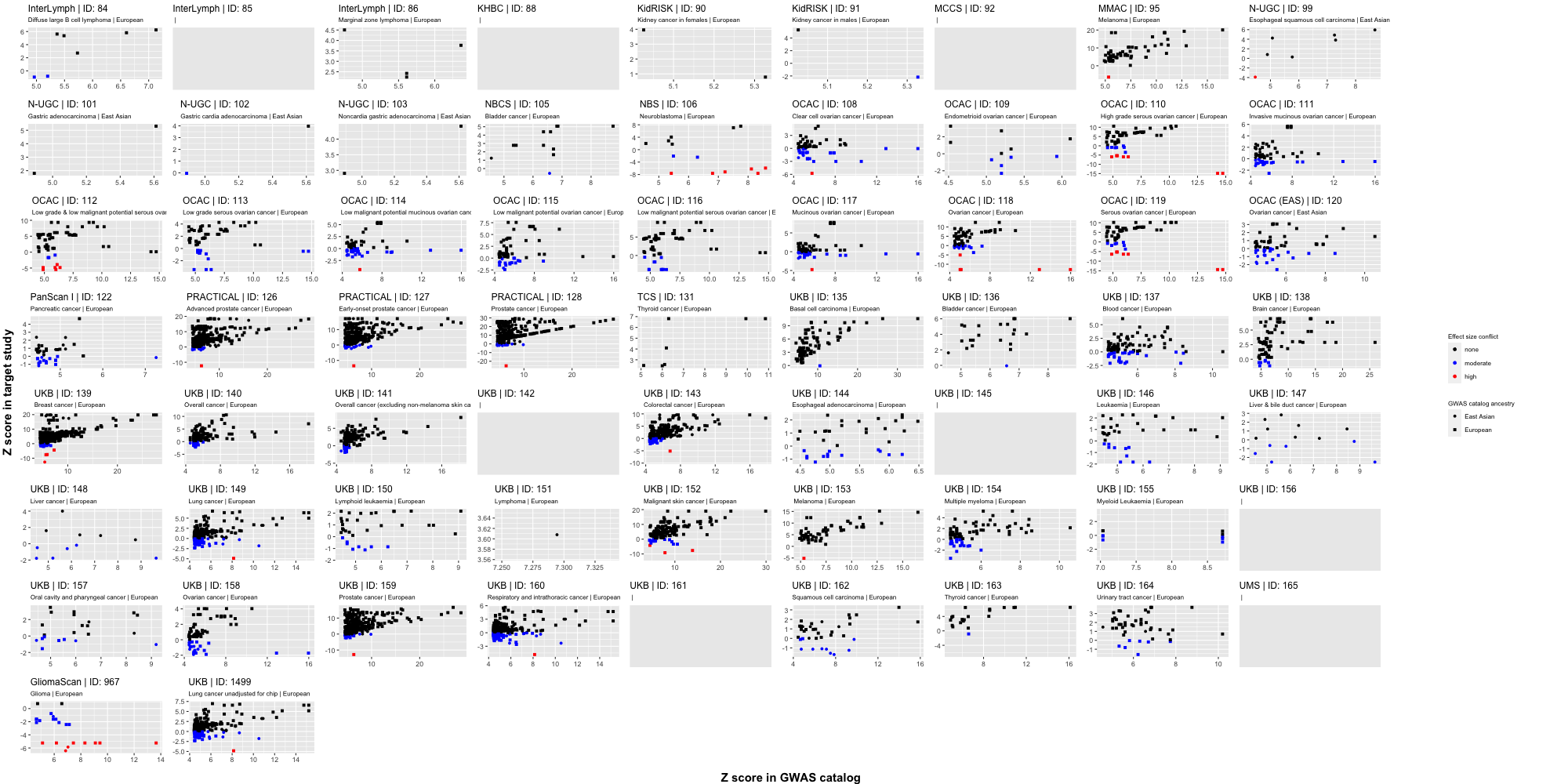


Study acronyms are explained in supplementary table 6. EBI, European Bioinformatics Institute; GWAS, genome-wide association study; NHGRI, National Human Genome Research Institute

Supplementary figure S14. Comparison of minor allele frequency between cancer datasets from European studies and the Cohorts for Heart and Aging Research in Genomic Epidemiology Consortium


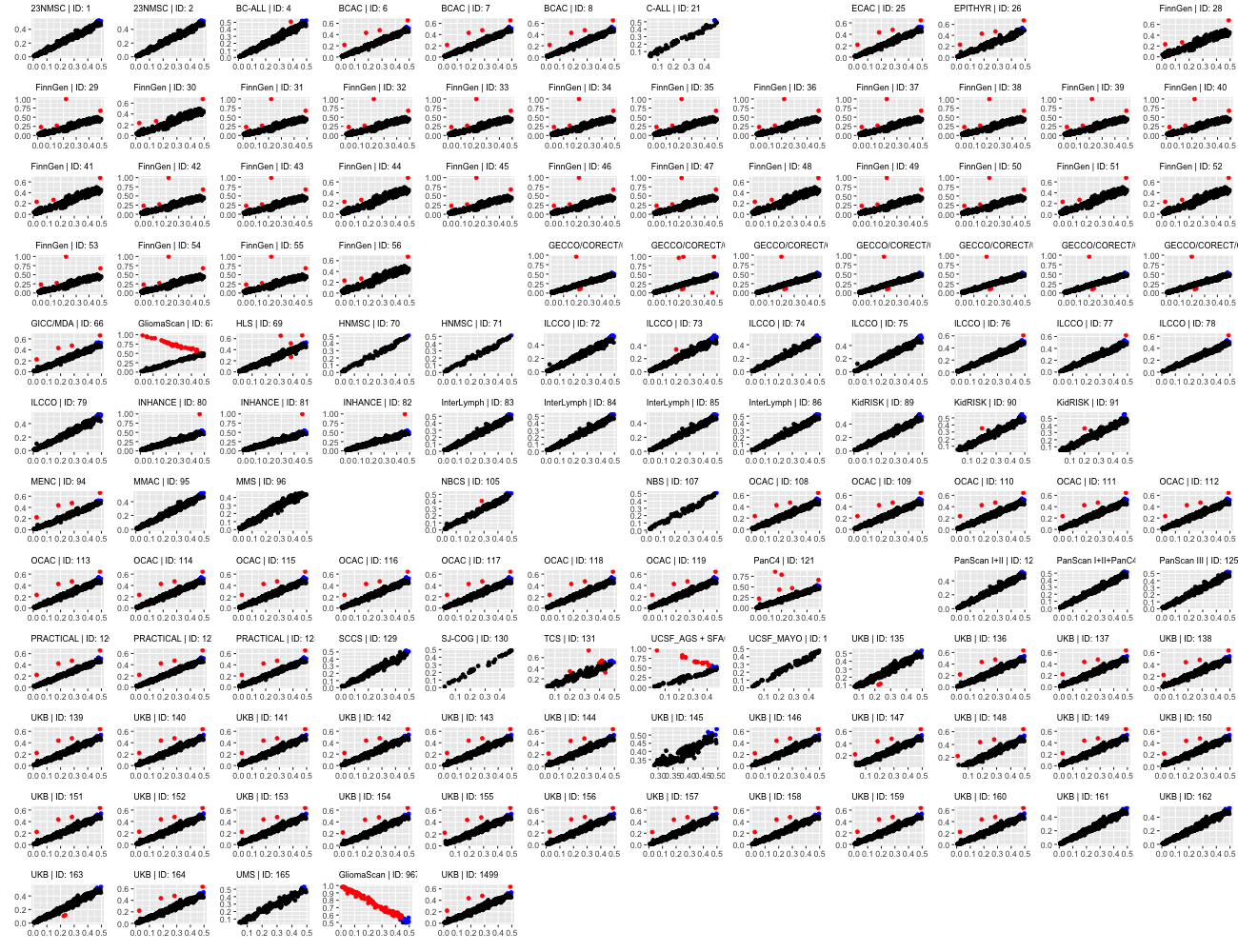


Red data points correspond to SNPs with high allele frequency conflicts, due to having an allele frequency that is greater than 0.58 (when it is expected to be less than 0.5) or to deviation from the reference allele frequency by more than 10 points. Study acronyms are explained in supplementary table 6.

Supplementary figure S15. Comparison of minor allele frequency between cancer datasets from East Asian studies and the Singapore Chinese Health Study


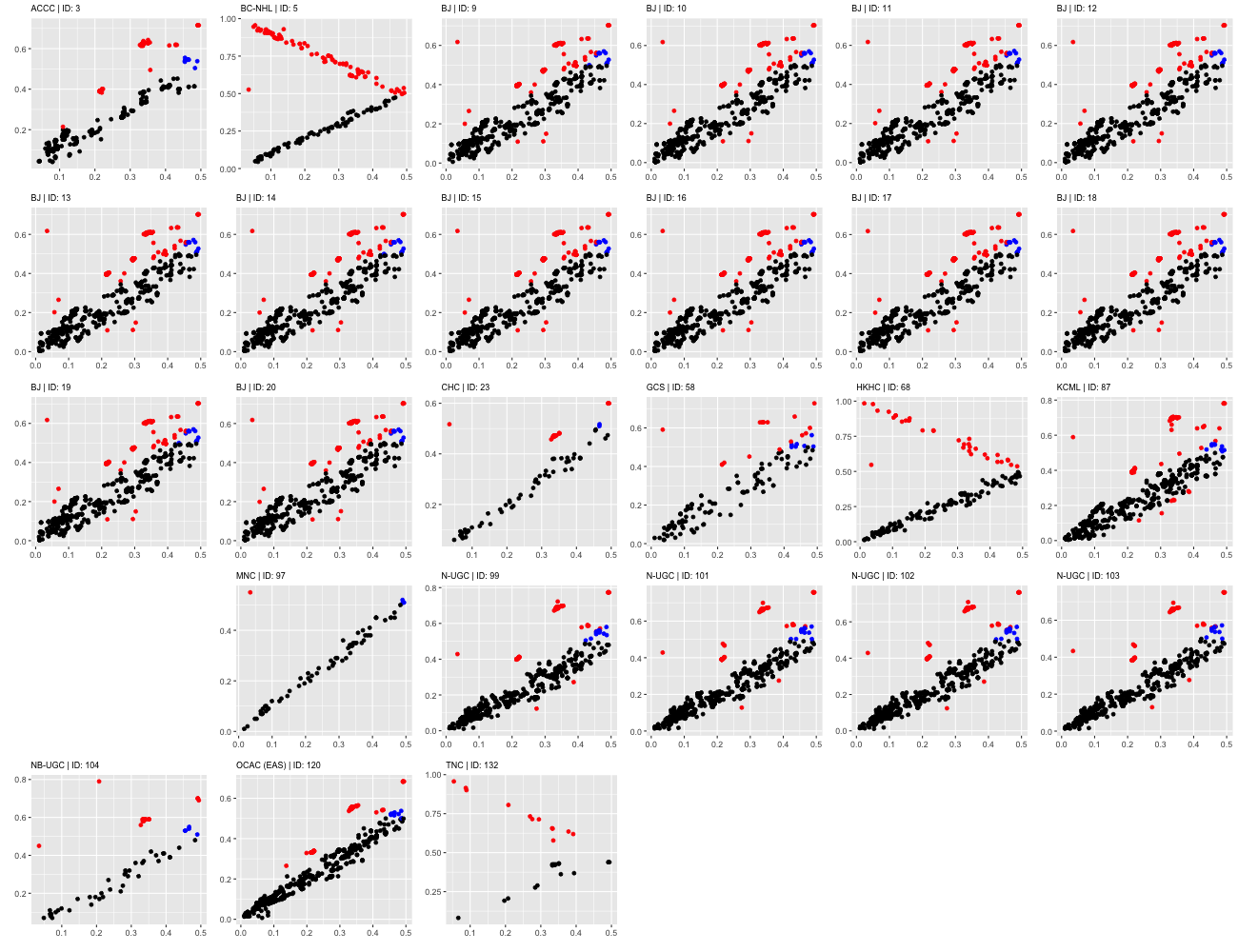


Red data points correspond to SNPs with high allele frequency conflicts, due to having an allele frequency that is greater than 0.58 (when it is expected to be less than 0.5) or to deviation from the reference allele frequency by more than 10 points. Study acronyms are explained in supplementary table 6.

Supplementary figure S16. Comparison of minor allele frequency between cancer datasets from East Asian studies and super populations from the 1000 genomes project


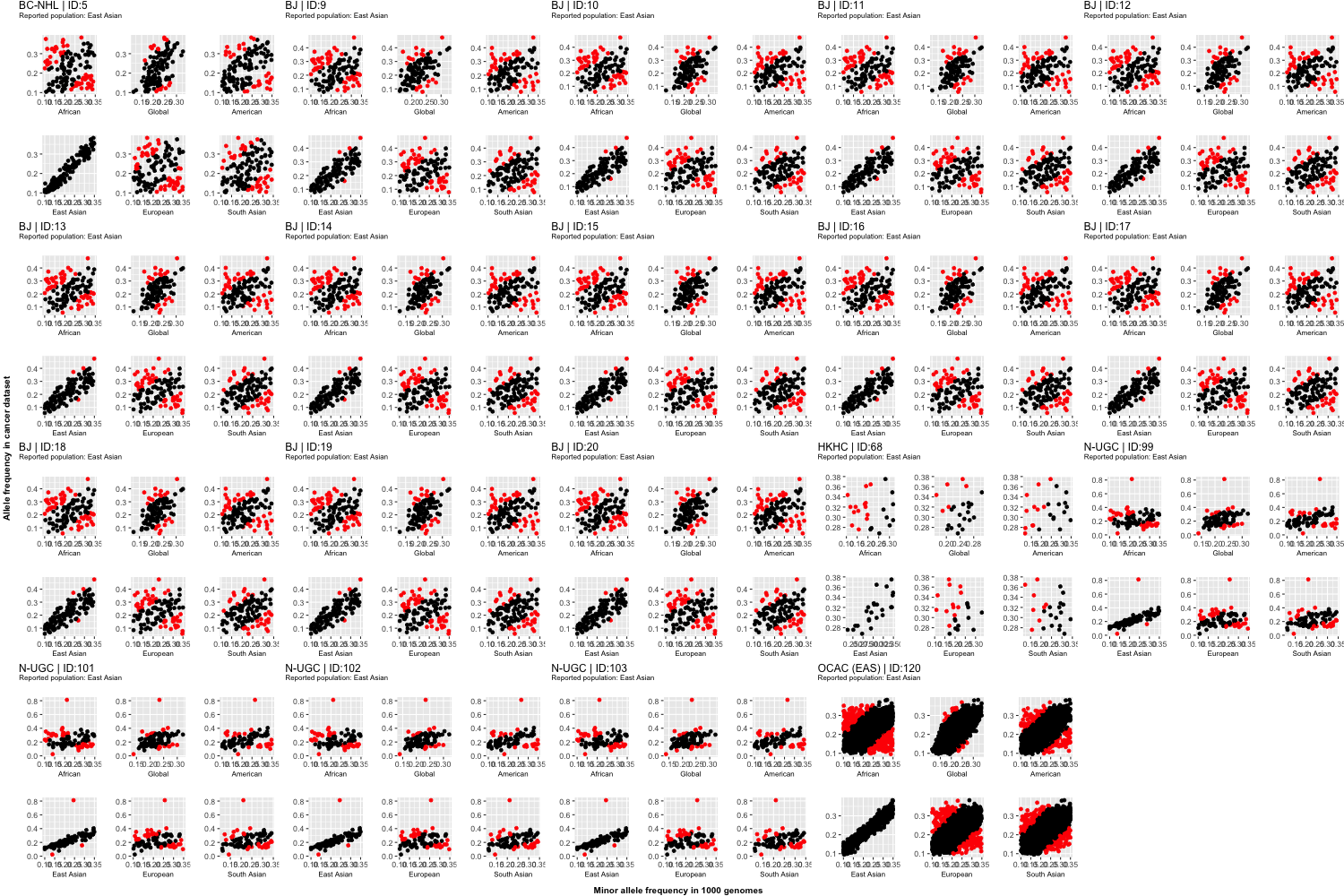


Each data point represents a single SNP. Red data points correspond to SNPs with high allele frequency conflicts, due to having an allele frequency that is greater than 0.58 (when it is expected to be less than 0.5) or to deviation from the reference allele frequency by more than 10 points. Study acronyms are explained in supplementary table 6.

Supplementary figure S17a. Comparison of minor allele frequency between cancer datasets [ID1 to ID45] from European studies and super populations from the 1000 genomes project


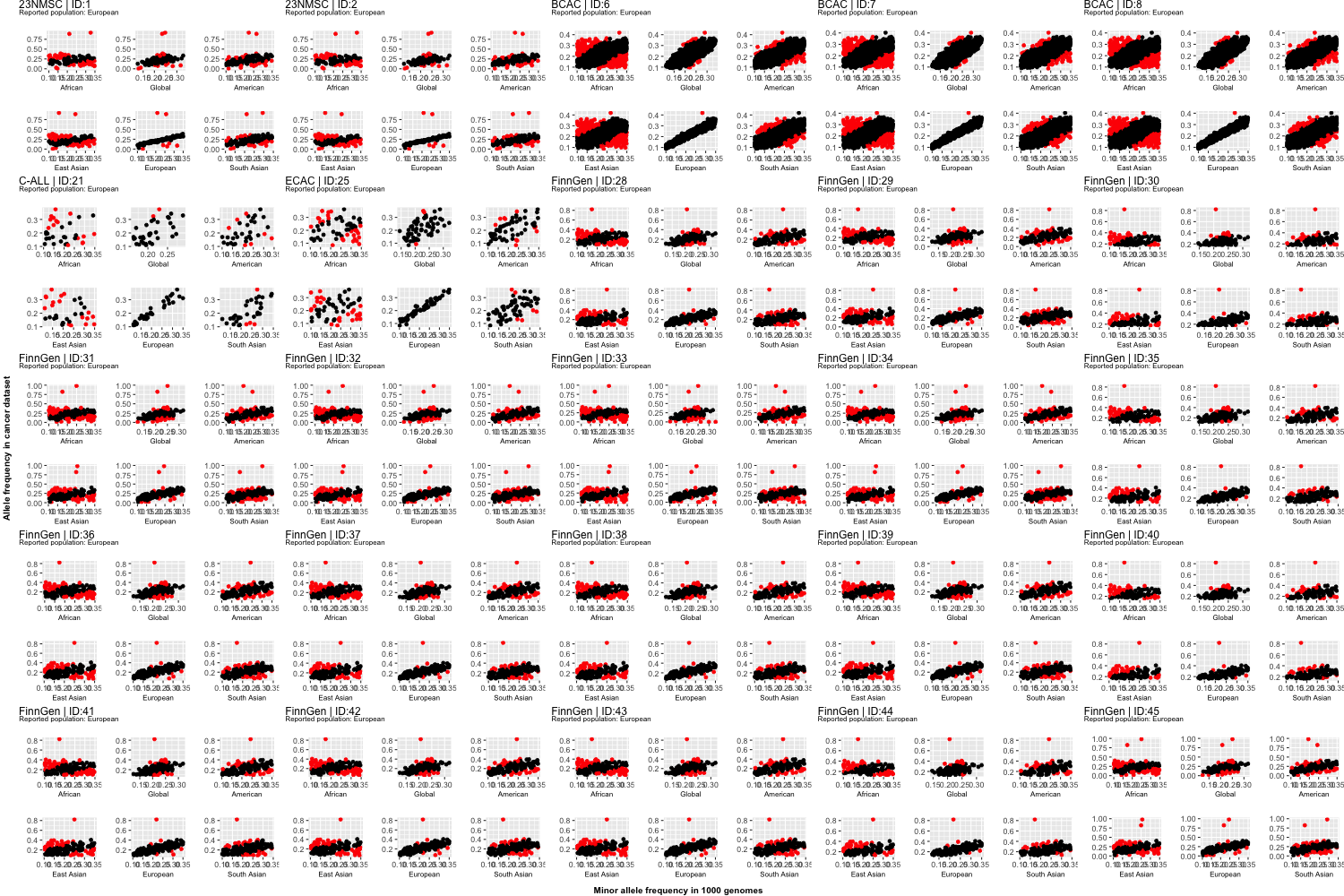


Each data point represents a single SNP. Red data points correspond to SNPs with high allele frequency conflicts, due to having an allele frequency that is greater than 0.58 (when it is expected to be less than 0.5) or to deviation from the reference allele frequency by more than 10 points. Study acronyms are explained in supplementary table 6.

Supplementary figure S17b. Comparison of minor allele frequency between cancer datasets [ID46 to ID86] from European studies and super populations from the 1000 genomes project


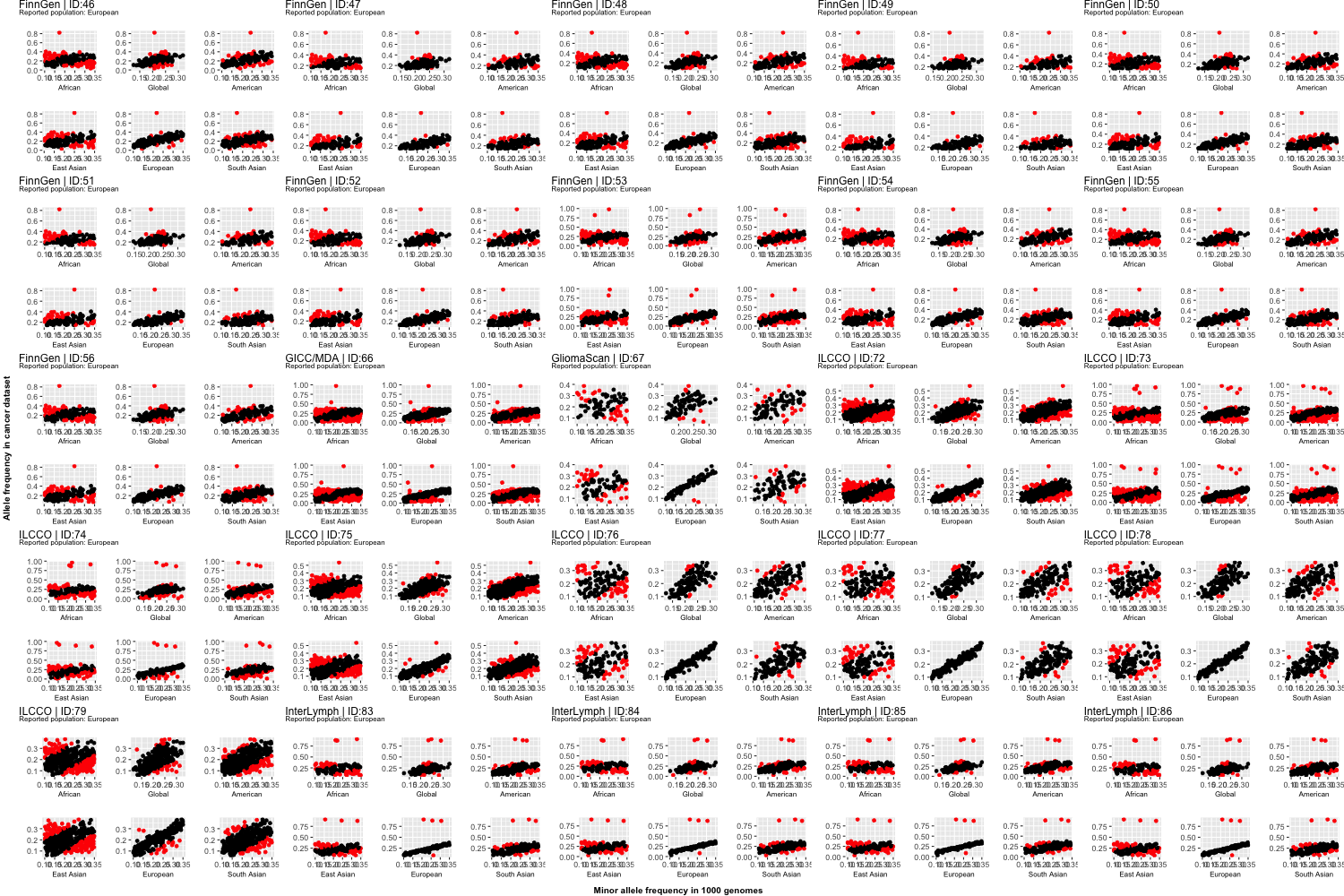


Each data point represents a single SNP. Red data points correspond to SNPs with high allele frequency conflicts, due to having an allele frequency that is greater than 0.58 (when it is expected to be less than 0.5) or to deviation from the reference allele frequency by more than 10 points. Study acronyms are explained in supplementary table 6.

Supplementary figure S17c. Comparison of minor allele frequency between cancer datasets [ID90 to ID139] from European studies and super populations from the 1000 genomes project


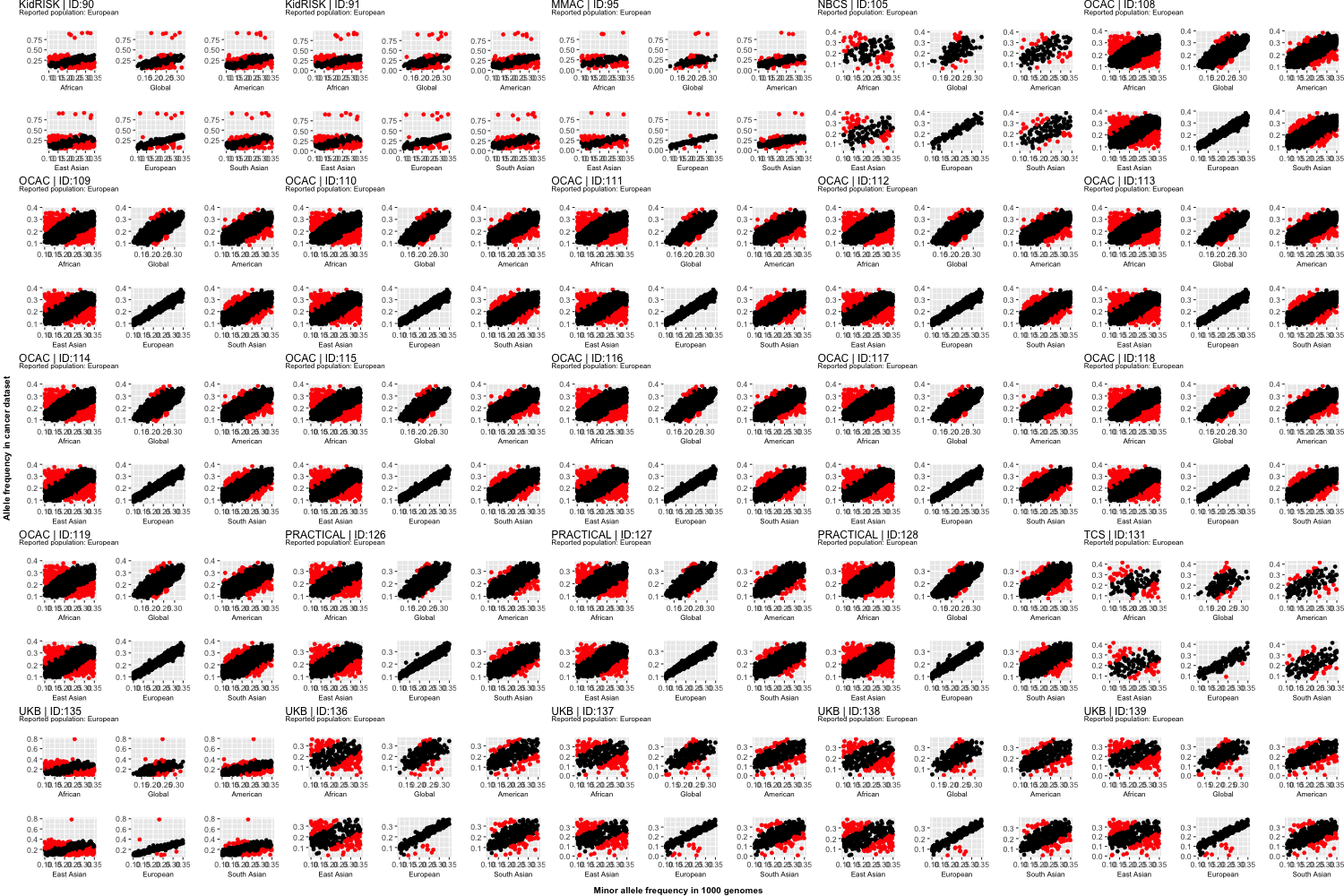


Each data point represents a single SNP. Red data points correspond to SNPs with high allele frequency conflicts, due to having an allele frequency that is greater than 0.58 (when it is expected to be less than 0.5) or to deviation from the reference allele frequency by more than 10 points. Study acronyms are explained in supplementary table 6.

Supplementary figure S17d. Comparison of minor allele frequency between cancer datasets [ID140 to ID1499] from European studies and super populations from the 1000 genomes project


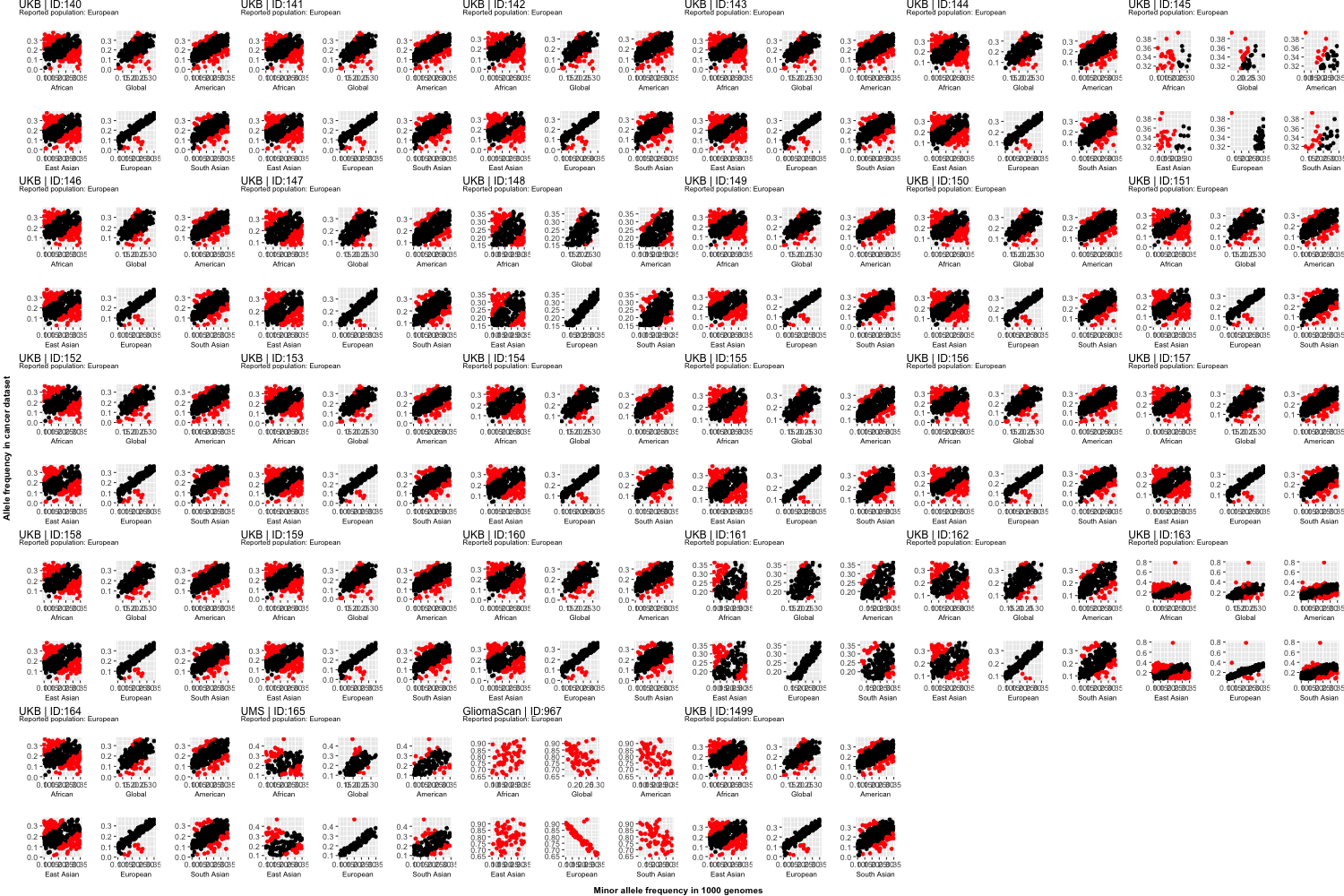


Each data point represents a single SNP. Red data points correspond to SNPs with high allele frequency conflicts, due to having an allele frequency that is greater than 0.58 (when it is expected to be less than 0.5) or to deviation from the reference allele frequency by more than 10 points. Study acronyms are explained in supplementary table 6.

Supplementary figure S18. Datasets with discrepancies between the expected log odds ratio and reported effect sizes

The plotted datasets correspond to slopes > 1.2 or <0.8 from models of the expected log odds ratio regressed on the reported effect size. Y axis corresponds to expected log odds ratios and X axis corresponds to reported log odds ratios


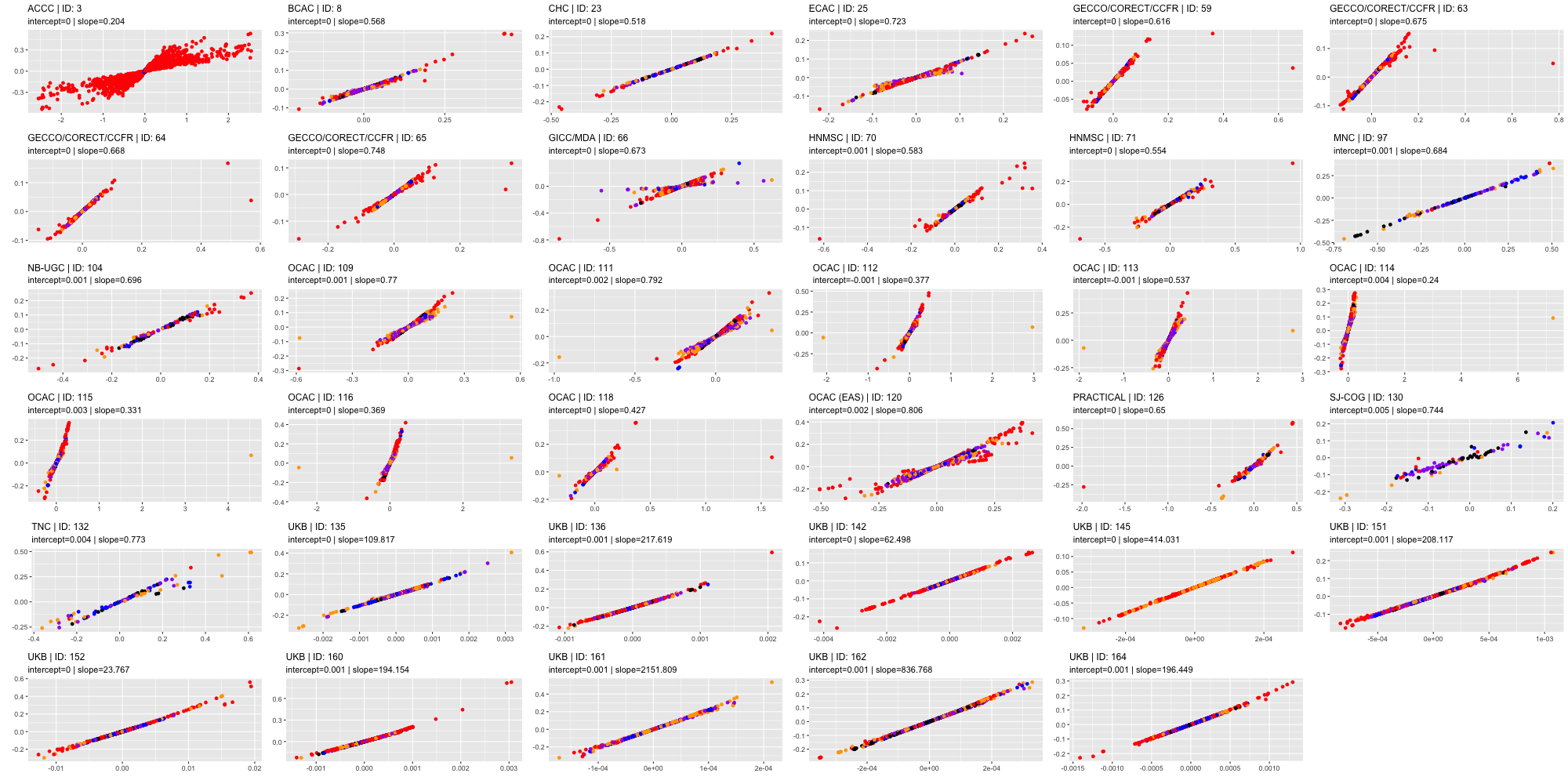

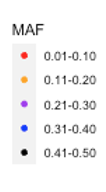


Study acronyms are explained in supplementary table 6.

Supplementary figure S19. Comparison of reported and expected log odds ratios in the ACCC dataset


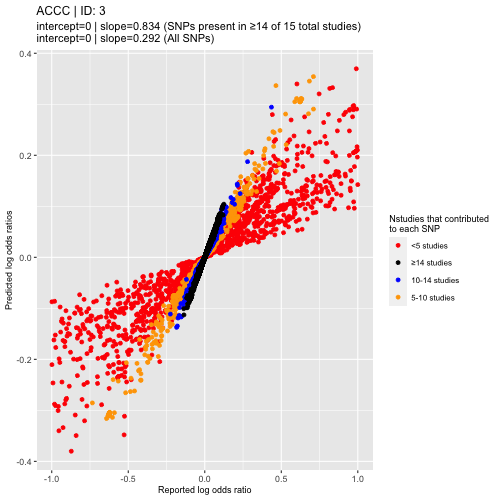


Slopes and intercepts were generated from models regressing the reported log odds ratio on the expected log odds ratio; ACCC, Asian Colorectal Cancer Consortium; SNP, single nucleotide polymorphism

Supplementary figure S20. Comparison of reported and expected log odds ratios in the GICC/MDA dataset


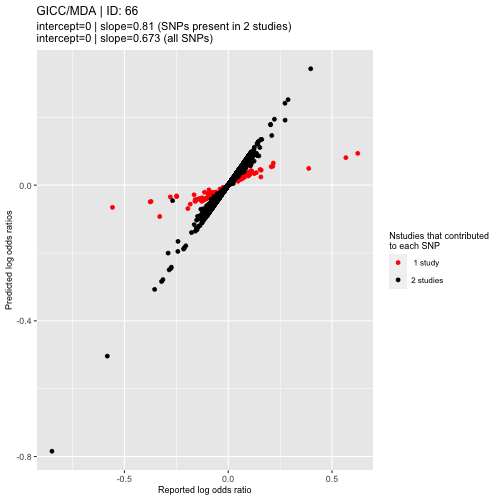


Slopes and intercepts were generated from models regressing the reported log odds ratio on the expected log odds ratio; GICC/MDA, Glioma International Case-Control Study/MD Anderson Cancer Center; SNP, single nucleotide polymorphism

Supplementary figure S21. Comparison of reported and expected log odds ratios in the GECCO* datasets


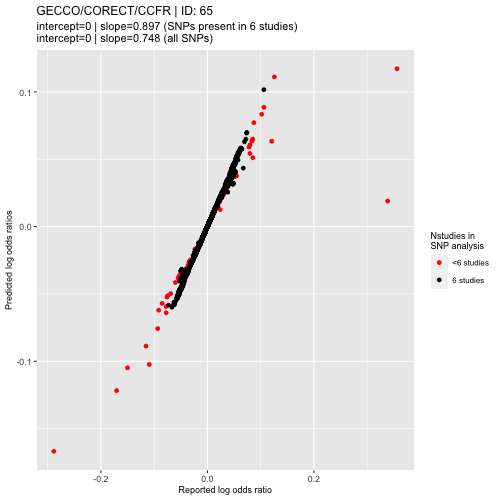

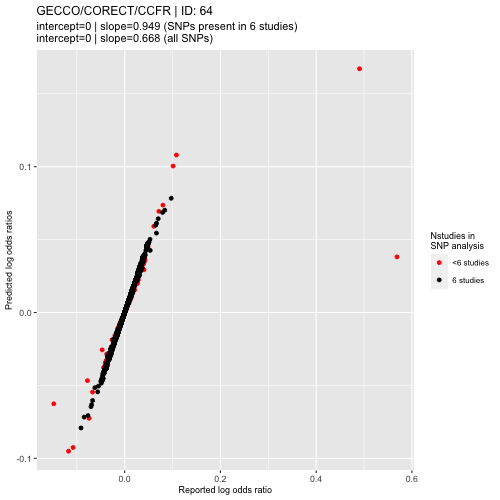

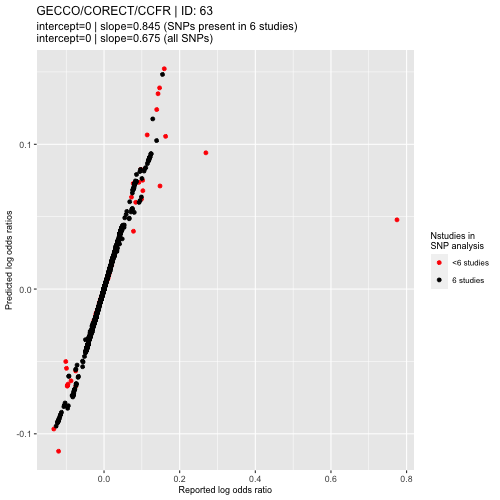

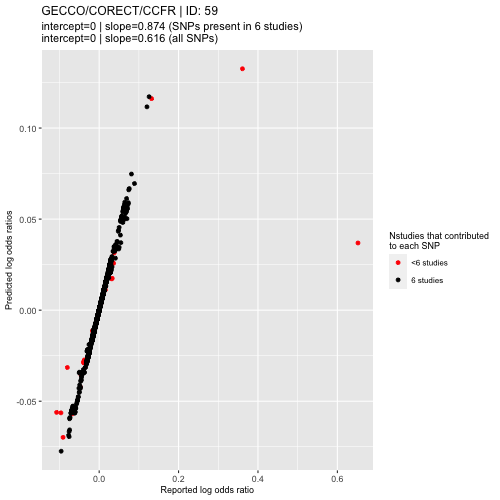


Slopes and intercepts were generated from models regressing the reported log odds ratio on the expected log odds ratio; *GECCO/CORECT/CCFR, Genetics and Epidemiology of Colorectal Cancer Consortium/Colorectal Transdisciplinary study/Colon Cancer Family Registry; SNP, single nucleotide polymorphism

Supplementary figure S22. Comparison of reported and expected log odds ratios in the HNMSC datasets


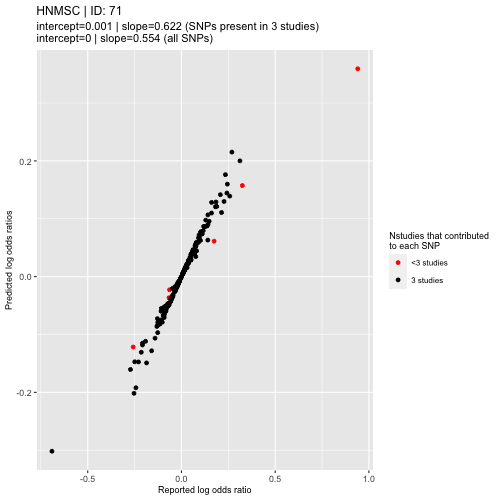

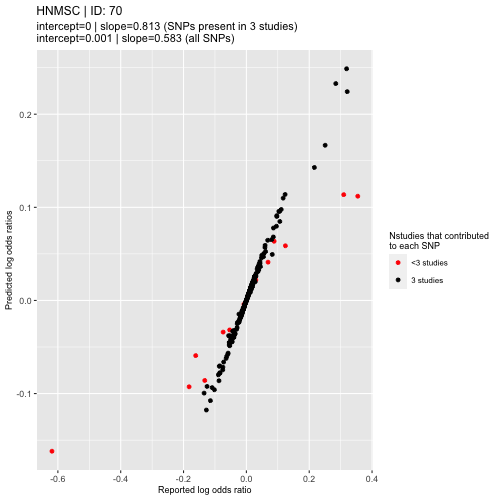


HNMSC, Harvard Non-Melanoma Skin Cancer Study; SNP, single nucleotide polymorphism

Supplementary figure S23. Deviations in reported from expected log odds ratios attributable to low imputation quality


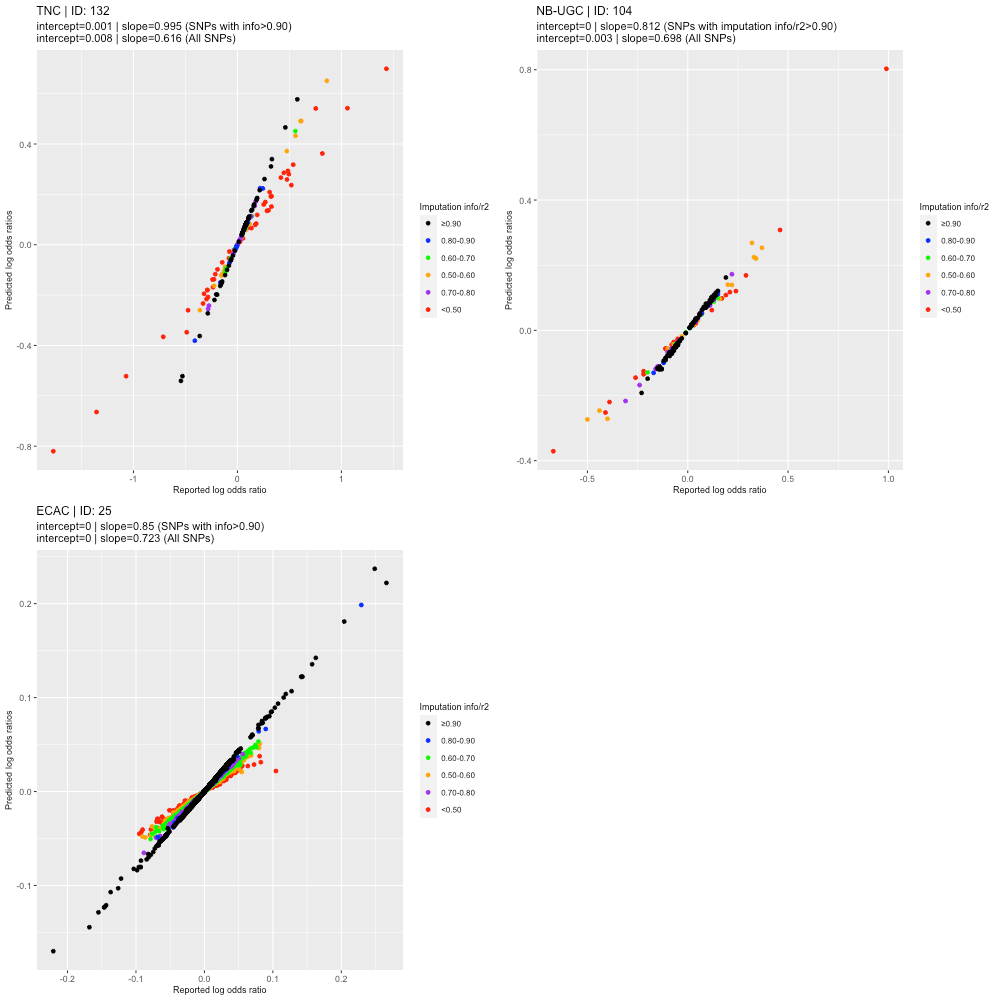


ECAC, Endometrial Cancer Association Consortium; NB-UGC, Nanjing+Beijing Upper Gastrointestinal Cancers Study; SNP, single nucleotide polymorphism; TNC, Taiwan Nasopharyngeal Carcinoma Study

**Supplementary figure S24.** Deviation between reported and expected log odds ratio and imputation quality


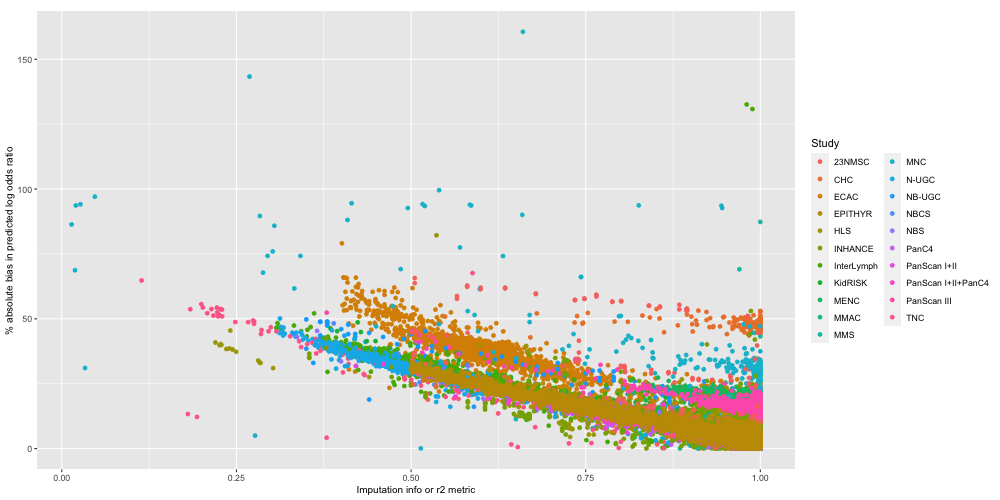


Study acronyms are explained in supplementary table 6.

**Supplementary figure S25.** Deviation between reported and expected log odds ratio and *P* value for deviations from Hardy Weinberg Equilibrium


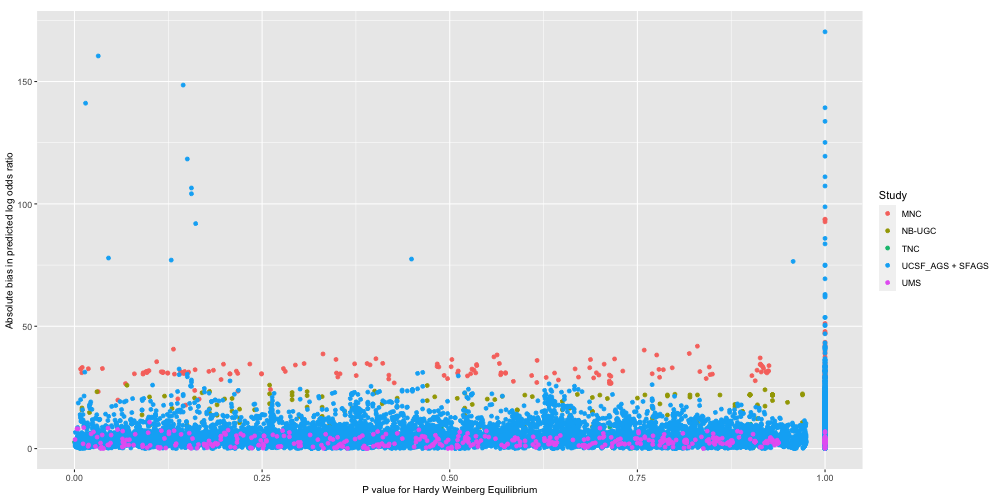


MNC, Malaysia Nasopharyngeal Carcinoma Study; NB-UGC, Nanjing+Beijing Upper Gastrointestinal Cancers Study;

TNC, Taiwan Nasopharyngeal Carcinoma Study; UCSF_AGS + SFAGS, UCSF Adult Glioma Study / San Francisco Adult Glioma Study

UMS, Uveal Melanoma Study

**Supplementary figure S26.** Deviation between reported and expected log odds ratio and *P* value for between study heterogeneity


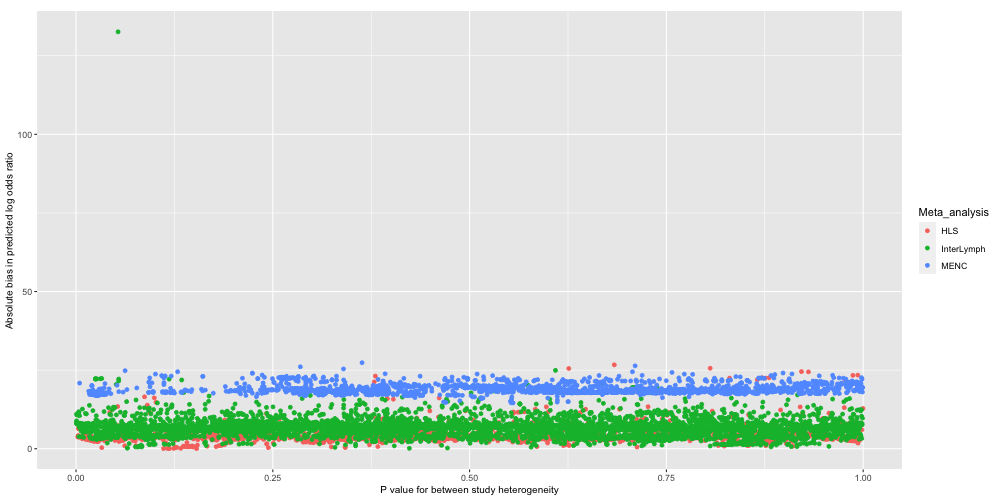


HLS, Hodgkin Lymphoma Study; MENC, The Meningioma Consortium

Supplementary figure S27. Quality control report for genetic summary data from a genome-wide association study of lung cancer in the ILCCO dataset (ID=74)


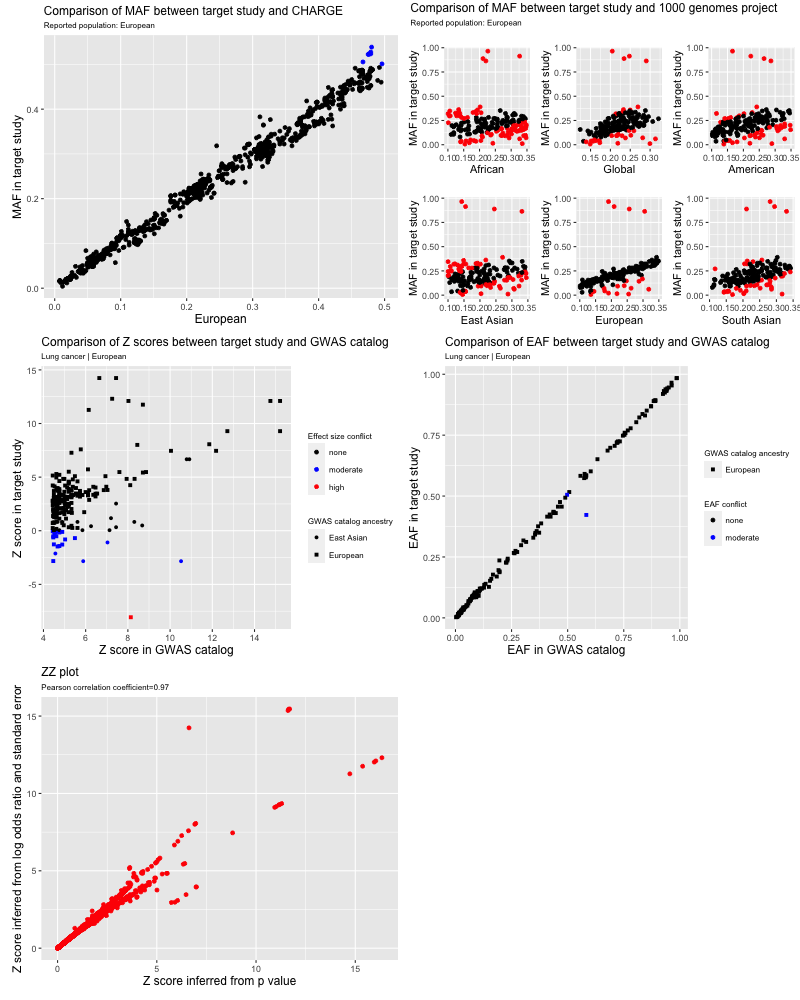


In the allele frequency subplots, each red data point corresponds to SNPs with high allele frequency conflicts, due to having an allele frequency that is greater than 0.58 (when it is expected to be less than 0.5) or to deviation from the reference allele frequency by more than 10 points. EAF, effect allele frequency; GWAS, genome-wide association study; ILCCO, The International Lung Cancer Consortium; MAF, minor allele frequency

Supplementary figure S28. Correlation between Z_p_ and Z_b_ scores within cancer datasets

Z_p_ = Z scores inferred from reported *P* values; Z_b_ = Z scores inferred from reported effect sizes and standard errors. Correlations less than 0.99 are highlighted in red


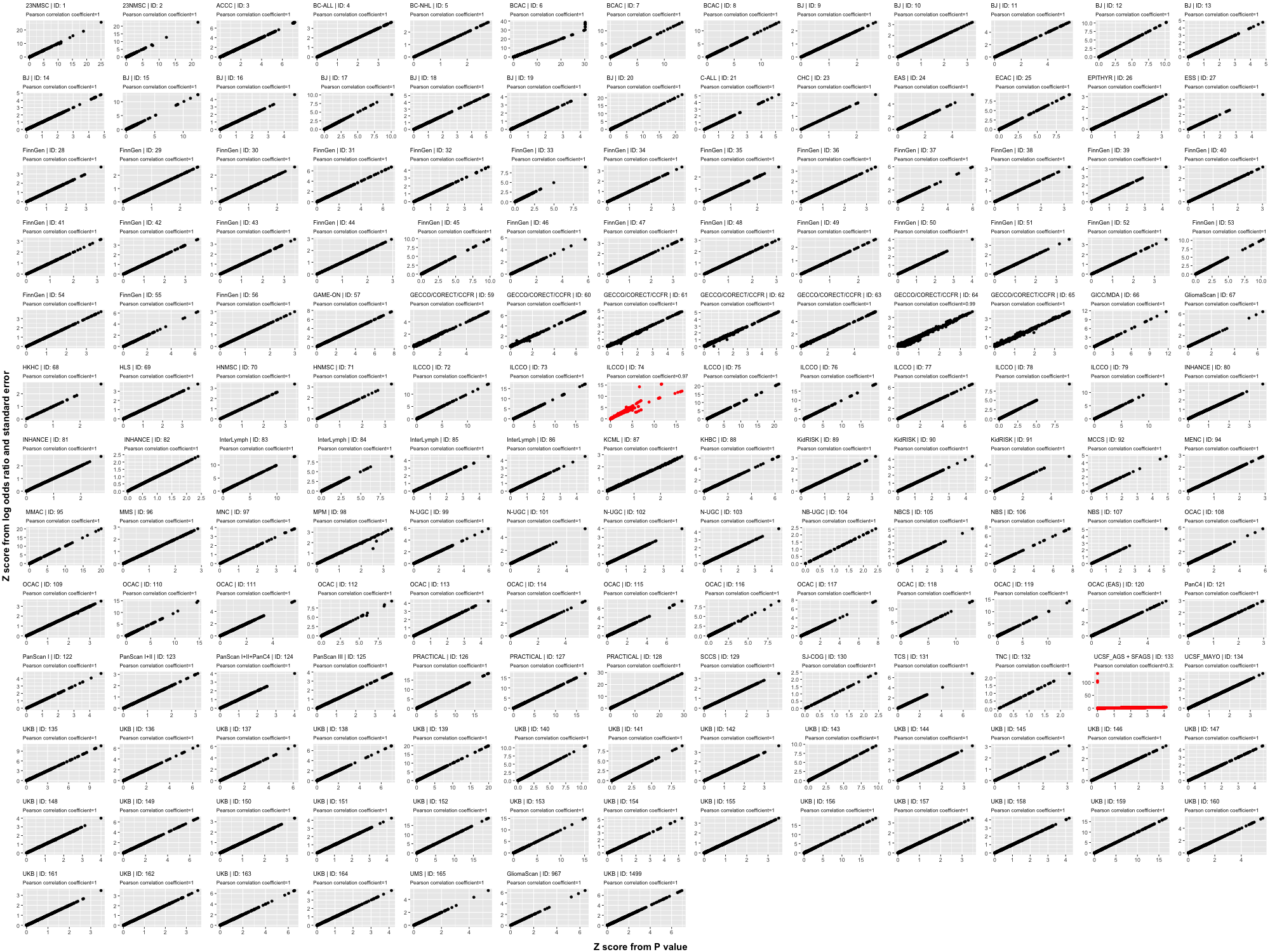


Study acronyms are explained in supplementary table 6.

Supplementary figure S29. Distribution of datasets by study

Study acronyms are explained in supplementary table 6.

Supplementary figure S30. Number of available cancer types by tissue or organ site

Supplementary figure S31. Max number of cases analysed by cancer site and study

Study acronyms are explained in supplementary table 6.
