# Supplementary material for: Design and quality control of large-scale two-sample Mendelian randomization studies
Source: Int J Epidemiol. Author manuscript; Available in PMC 2024 Jan 8. (PMC10555669; doi:10.1093/ije/dyad018)
Supplement: Supplementary material [file EMS190936-supplement-Supplementary_material.docx]

# Example of data sharing instructions

| Types of data needed |
| --- |
| Please supply the following summary data and supporting information: |
| 1. Natural log odds ratio for each SNP from an additive genetic model |
| 2. Standard error of the log odds ratio |
| 3. Effect allele (i.e. the coded allele in the additive genetic model) |
| 4. Non-effect allele |
| 5. Effect allele frequency |
| 6. A PubMed identifier (PMID) for a paper we can use as reference for the data (if published) |
| 7. Number of cases and controls in the GWAS analysis |
| 8. Confirmation of whether the data have been GC corrected |
| 9. Genome build and reference strand |
|  |
| It would also be helpful if you could include the following metrics of SNP genotype quality: |
| 10. P values for Hardy–Weinberg equilibrium |
| 11. P values / Cochran's Q test for between study heterogeneity (if your study is a meta-analysis) |
| 12. Metrics of imputation quality, such as info / r2 scores (for imputed SNPs) |
|  |
| A document describing the characteristics of your samples and the statistical analysis: |
| 13. Mean (standard deviation) of age in controls and cases |
| 14. Number of, or percentage, male participants in controls and cases |
| 15. Ethnicity of study participants (e.g. East Asian, European, etc) |
| 16. Covariates included in the GWAS regression model (e.g. age, sex, principal components, etc) |
| 17. Genomic control or inflation factor |
| 18. Genotype and sample QC procedures (e.g. exclusions based on HWE, call rates, etc) |

# Method for predicting log odds ratios

We predicted the log odds ratio for each SNP from its Z score (ratio of the reported log odds ratio to its standard error), reported minor allele frequency and the reported number of cases and controls^17^. The method has three main steps: first the overall odds of the cancer outcome in the study are estimated (number of cases divided by total sample size); second, the number of participants with 0, 1 or 2 copies of the minor allele are estimated; and third the log odds ratio is found that gives the same Z score given the number of cases and controls, the odds, effect direction and minor allele frequency. For the third step, a million values of Z for log odds ratios between 0 and 1 (or -1, depending on the effect direction of Z) are simulated. Then the minimum difference between the observed and estimated Z scores is selected. If the minimum score is at the maximum value of Z, the next million values are simulated and so on until the first minimum is reached. The method assumes 1) that covariates in the original genome-wide association study (GWAS) had a minimal effect on the actual log odds ratio and standard error and 2) that SNPs are in Hardy-Weinberg equilibrium. In simulations assuming Hardy-Weinberg equilibrium, the method slightly underestimated the true log odd ratio and the degree of bias was small: the median percentage difference between the true and predicted log odds ratio was -0.02% (interquartile range: -0.52% to 0.56%) with a slope of 0.95 in a model of the predicted log odds ratio regressed on the true log odds ratio. When restricting to log odds ratios between -1 and 1, which should contain the vast majority of log odds ratios observed in GWAS, the slope was 0.99, with a median percentage difference between the predicted and true log odds ratio of -0.05% (interquartile range -0.42% to 0.34%). Further details can be found in Harrison 2020^17^.

# Estimation of total number of cases and controls

Application of the quality control pipeline to the cancer GWAS lead to the retention of 160 datasets from 51 studies, consortia or biobanks (**Supplementary Figure S29**). Given that many cancer analyses and datasets were generated from the same biobank or consortium, there was potential to over-estimate the total number of cases and controls. To estimate the number of non-overlapping cases and controls in the FAMRC, we retained a single cancer per consortium or biobank, retaining the cancer that had the largest number of cases. We further excluded the GAME-ON consortium (FAMRC ID=57), which overlaps with consortia for breast, prostate, lung, ovarian and colorectal cancer, and excluded a pancreatic cancer GWAS meta-analysis (FAMRC ID = 124), which overlapped with various individual GWAS of pancreatic cancer (FAMRC IDs= 121, 122,123 and 125). These steps led to an estimate of 566,665 cases and 1,622,374 controls.

# Acknowledgements and individual cancer study descriptions

## 23andMe Non-Melanoma Skin Cancer Study

# 23andMe participants provided informed consent and participated in the research online, under a protocol approved by the external AAHRPP-accredited IRB, Ethical & Independent Review Services (E&I Review). The full GWAS summary statistics for the 23andMe discovery data set will be made available through 23andMe to qualified researchers under an agreement with 23andMe that protects the privacy of the 23andMe participants. Please visit <https://research.23andme.com/collaborate/#dataset-access/> for more information and to apply to access the data.

## International Lung Cancer Consortium

The authors gratefully acknowledge the following investigators and contributors to the International Lung Cancer Consortium: Demetrios Albanes (ATBC), Stephen Lam (Canadian screening study), Adonina Tardon (CAPUA STUDY), Chu Chen (CARET), Stig E. Bojesen (Copenhagen study), Maria Teresa Landi (EAGLE), Mattias Johansson (EPIC: European Prospective Investigation into Cancer and Nutrition), Angela Risch (German Lung Cancer Study - DKFZ), Heike Bickeböller (German Lung Cancer Study -LUCY), H-Erich Wichmann (German Lung Cancer Study - LUCY), David Christiani (Harvard Lung Cancer Study), Gadi Rennert (Israel study), Susanne Arnold (Kentucky LCRI-DOD), Paul Brennan and James McKay (L2-IARC), John K. Field (Liverpool Lung Project), Sanjay S. Shete (MDACC), Loic Le Marchand (MEC), Olle Melander (MDCS: The Malmö Diet and Cancer Study), Hans Brunnström (MDCS: The Malmö Diet and Cancer Study), Geoffrey Liu (MSH-PMH, Canadian Screening studies), Rayjean J. Hung (MSH-PMH), Angeline Andrew (NELCS), Lambertus A. Kiemeney (Nijmegen), Shan Zienolddiny-Narui (Norway), Kjell Grankvist (NSHDS: Northern Sweden Health and Disease Study), Mikael Johansson (NSHDS: Northern Sweden Health and Disease Study), Neil Caporaso (PLCO), Angie Cox (ReSoLucent), Philip Lazarus (Tampa Lung Cancer Study), Matthew B. Schabath (Total Lung Cancer (TLC): Molecular Epidemiology of Lung Cancer Survival), Melinda C. Aldrich (Vanderbilt Lung Cancer Study - BioVU).

# References

1. Zheng J-S, Hu X-J, Zhao Y-M, Yang J, Li D. Intake of fish and marine n-3 polyunsaturated fatty acids and risk of breast cancer: meta-analysis of data from 21 independent prospective cohort studies. *BMJ*. 2013;346(June 2013):f3706. doi:10.1136/bmj.f3706

2. Wang J, Zhang Y, Zhao L. Omega-3 PUFA intake and the risk of digestive system cancers: A meta-analysis of observational studies. *Medicine (Baltimore)*. 2020;99(19):e20119. doi:10.1097/MD.0000000000020119

3. Sadeghi A, Shab-Bidar S, Parohan M, Djafarian K. Dietary Fat Intake and Risk of Ovarian Cancer: A Systematic Review and Dose–Response Meta-Analysis of Observational Studies. *Nutr Cancer*. 2019;71(6):939-953. doi:10.1080/01635581.2019.1595049

4. Qiu W, Lu H, Qi Y, Wang X. Dietary fat intake and ovarian cancer risk: A meta-analysis of epidemiological studies. *Oncotarget*. 2016;7(24):37390-37406. doi:10.18632/oncotarget.8940

5. Yang JJ, Yu D, Takata Y, et al. Dietary fat intake & lung cancer risk: A pooled analysis. *J Clin Oncol*. 2017;35(26):3055-3064. doi:10.1200/JCO.2017.73.3329

6. Han J, Jiang Y, Liu X, et al. Dietary Fat Intake and Risk of Gastric Cancer: A Meta-Analysis of Observational Studies. Chiu C-J, ed. *PLoS One*. 2015;10(9):e0138580. doi:10.1371/journal.pone.0138580

7. Ruan L, Cheng S-P, Zhu Q-X. Dietary Fat Intake and the Risk of Skin Cancer: A Systematic Review and Meta-Analysis of Observational Studies. *Nutr Cancer*. 2020;72(3):398-408. doi:10.1080/01635581.2019.1637910

8. Xia H, Ma S, Wang S, Sun G. Meta-analysis of saturated fatty acid intake and breast cancer risk. *Med (United States)*. 2015;94(52). doi:10.1097/MD.0000000000002391

9. Aglago EK, Huybrechts I, Murphy N, et al. Consumption of Fish and Long-chain n-3 Polyunsaturated Fatty Acids Is Associated With Reduced Risk of Colorectal Cancer in a Large European Cohort. *Clin Gastroenterol Hepatol*. 2020;18(3):654-666.e6. doi:10.1016/j.cgh.2019.06.031

10. Wu S, Liang J, Zhang L, Zhu X, Liu X, Miao D. Fish consumption and the risk of gastric cancer: systematic review and meta-analysis. *BMC Cancer*. 2011;11(1):26. doi:10.1186/1471-2407-11-26

11. Kim M, Park K. Dietary Fat Intake and Risk of Colorectal Cancer: A Systematic Review and Meta-Analysis of Prospective Studies. *Nutrients*. 2018;10(12):1963. doi:10.3390/nu10121963

12. Chua ME, Sio MCD, Sorongon MC, Dy JS. Relationship of dietary intake of omega-3 and omega-6 Fatty acids with risk of prostate cancer development: a meta-analysis of prospective studies and review of literature. *Prostate Cancer*. 2012;2012:826254. doi:10.1155/2012/826254

13. Alexander DD, Bassett JK, Weed DL, Barrett EC, Watson H, Harris W. Meta-analysis of long-chain omega-3 polyunsaturated fatty acids (LCω-3PUFA) and prostate cancer. *Nutr Cancer*. 2015;67(4):543-554. doi:10.1080/01635581.2015.1015745

14. Liu J, Li X, Hou J, Sun J, Guo N, Wang Z. Dietary Intake of N-3 and N-6 Polyunsaturated Fatty Acids and Risk of Cancer: Meta-Analysis of Data from 32 Studies. *Nutr Cancer*. June 2020:1-13. doi:10.1080/01635581.2020.1779321

15. Sakai M, Kakutani S, Horikawa C, et al. Arachidonic acid and cancer risk: a systematic review of observational studies. *BMC Cancer*. 2012;12(1):606. doi:10.1186/1471-2407-12-606

16. Zhao J, Lyu C, Gao J, et al. Dietary fat intake and endometrial cancer risk. *Medicine (Baltimore)*. 2016;95(27):e4121. doi:10.1097/MD.0000000000004121

17. Harrison S. Estimating an Odds Ratio from a GWAS only reporting the P value – Sean Harrison: Blog. https://seanharrisonblog.com/2020/04/11/estimating-an-odds-ratio-from-a-gwas-only-reporting-the-p-value/. Published 2020. Accessed December 23, 2020.

18. Bowden J, Del Greco M F, Minelli C, Davey Smith G, Sheehan N, Thompson J. A framework for the investigation of pleiotropy in two-sample summary data Mendelian randomization. *Stat Med*. 2017;36(11):1783-1802. doi:10.1002/sim.7221

19. Bowden J, Davey Smith G, Burgess S. Mendelian randomization with invalid instruments: effect estimation and bias detection through Egger regression. *Int J Epidemiol*. 2015;44(2):512-525. doi:10.1093/ije/dyv080

20. Bowden J, Davey Smith G, Haycock PC, Burgess S. Consistent estimation in Mendelian randomization with some invalid instruments using a weighted median estimator. *Genet Epidemiol*. http://www.mrc-bsu.cam.ac.uk/wp-content/uploads/SimpleWeightedMedian.pdf.

21. Burgess S, Freitag DF, Khan H, Gorman DN, Thompson SG. Using multivariable Mendelian randomization to disentangle the causal effects of lipid fractions. *PLoS One*. 2014;9(10):e108891. doi:10.1371/journal.pone.0108891

22. Hartwig FP, Davey Smith G, Bowden J. Robust inference in summary data Mendelian randomization via the zero modal pleiotropy assumption. *Int J Epidemiol*. 2017;46(6):1985-1998. doi:10.1093/ije/dyx102

23. Sterne JAC, Sutton AJ, Ioannidis JPA, et al. Recommendations for examining and interpreting funnel plot asymmetry in meta-analyses of randomised controlled trials. *BMJ*. 2011;343(7818). doi:10.1136/bmj.d4002

24. Giambartolomei C, Vukcevic D, Schadt EE, et al. Bayesian Test for Colocalisation between Pairs of Genetic Association Studies Using Summary Statistics. Williams SM, ed. *PLoS Genet*. 2014;10(5):e1004383. doi:10.1371/journal.pgen.1004383
